# Altered Epigenetic Marks and Gene Expression in Fetal Brain, and Postnatal Behavioural Disorders, Following Prenatal Exposure of *Ogg1* Knockout Mice to Saline or Ethanol

**DOI:** 10.3390/cells12182308

**Published:** 2023-09-19

**Authors:** Shama Bhatia, David Bodenstein, Ashley P. Cheng, Peter G. Wells

**Affiliations:** 1 Department of Pharmaceutical Sciences, Faculty of Pharmacy, University of Toronto, Toronto, ON M5S 3M2, Canada; shama.bhatia@mail.utoronto.ca (S.B.); ashley.cheng@mail.utoronto.ca (A.P.C.); 2 Centre for Pharmaceutical Oncology, Faculty of Pharmacy, University of Toronto, Toronto, ON M5S 3M2, Canada; 3 Department of Pharmacology & Toxicology, Faculty of Medicine, University of Toronto, Toronto, ON M5S 1A8, Canada; d.bodenstein@mail.utoronto.ca

**Keywords:** alcohol (ethanol, EtOH), DNA damage and repair, DNA methylation, epigenetic changes, fetal alcohol spectrum disorders (FASDs), histone methylation and acetylation, neurodevelopmental disorders, oxoguanine glycosylase 1 (OGG1), reactive oxygen species (ROS)

## Abstract

Oxoguanine glycosylase 1 (OGG1) is widely known to repair the reactive oxygen species (ROS)-initiated DNA lesion 8-oxoguanine (8-oxoG), and more recently was shown to act as an epigenetic modifier. We have previously shown that saline-exposed *Ogg1* −/− knockout progeny exhibited learning and memory deficits, which were enhanced by in utero exposure to a single low dose of ethanol (EtOH) in both *Ogg1* +/+ and −/− progeny, but more so in *Ogg1* −/− progeny. Herein, OGG1-deficient progeny exposed in utero to a single low dose of EtOH or its saline vehicle exhibited OGG1- and/or EtOH-dependent alterations in global histone methylation and acetylation, DNA methylation and gene expression (*Tet1* (Tet Methylcytosine Dioxygenase 1)*, Nlgn3* (Neuroligin 3)*, Hdac2* (Histone Deacetylase 2)*, Reln* (Reelin) and *Esr1* (Estrogen Receptor 1)) in fetal brains, and behavioural changes in open field activity, social interaction and ultrasonic vocalization, but not prepulse inhibition. OGG1- and EtOH-dependent changes in *Esr1* and *Esr2* mRNA and protein levels were sex-dependent, as was the association of *Esr1* gene expression with gene activation mark histone H3 lysine 4 trimethylation (H3K4me3) and gene repression mark histone H3 lysine 27 trimethylation (H3K27me3) measured via ChIP-qPCR. The OGG1-dependent changes in global epigenetic marks and gene/protein expression in fetal brains, and postnatal behavioural changes, observed in both saline- and EtOH-exposed progeny, suggest the involvement of epigenetic mechanisms in developmental disorders mediated by 8-oxoG and/or OGG1. Epigenetic effects of OGG1 may be involved in ESR1-mediated gene regulation, which may be altered by physiological and EtOH-enhanced levels of ROS formation, possibly contributing to sex-dependent developmental disorders observed in *Ogg1* knockout mice. The OGG1- and EtOH-dependent associations provide a basis for more comprehensive mechanistic studies to determine the causal involvement of oxidative DNA damage and epigenetic changes in ROS-mediated neurodevelopmental disorders.

## 1. Introduction

Oxoguanine glycosylase 1 (OGG1) repairs 8-oxoguanine (8-oxoG), the major DNA lesion caused by reactive oxygen species (ROS) [1,2]. 8-OxoG is a mutagenic lesion potentially involved in carcinogenesis, and more recently has been shown to contribute to developmental disorders, likely via epigenetic (non-mutagenic) mechanisms [3,4,5]. ROS are involved in many processes that are essential for life, yet even normal levels cause measurable oxidative DNA damage including 8-oxoG formation [6], which is further increased by ROS-enhancing drugs like ethanol (EtOH) [3,4,5]. In humans, exposure of the embryo or fetus to EtOH during pregnancy results in an array of morphological and functional fetal alcohol spectrum disorders (FASDs) via multiple mechanisms likely including ROS-initiated oxidative stress and oxidative DNA damage [3,4]. Using *Ogg1* knockout mice, our lab has previously found that *Ogg1* −/− progeny exposed in utero to saline exhibited learning and memory deficits, which were further enhanced by in utero exposure to a single low dose of EtOH on gestational day (GD) 17 in both *Ogg1* +/+ and −/− littermates, with *Ogg1* −/− progeny being most severely affected [7]. This OGG1-dependent susceptibility to altered learning and memory caused by both physiological and EtOH-enhanced levels of ROS formation suggests a role for oxidative DNA damage in the pathogenic mechanism. Aside from the role of OGG1 in DNA repair, recent studies have revealed multiple epigenetic and other non-mutational mechanisms by which 8-oxoG and OGG1 may regulate gene transcription and signal transduction [8,9]. We recently found that untreated OGG1-deficient progeny exhibit a sex-dependent enhancement in DNA strand breaks, decreased DNA methylation levels and disorders of brain function compared to *Ogg1* +/+ littermates [10].

Over 3% of Canadian children aged 5–14 are estimated to have a neurodevelopmental disorder [11], while the incidence of FASDs in Canada and the USA is at least 1%, but may be as high as 3.1–9.8% in some regions [12,13]. EtOH causes several epigenetic modifications including changes in histone acetylation and methylation as well as DNA methylation, which have been associated with its embryopathic mechanism [14,15,16]. To determine if ROS-initiated DNA damage may cause neurodevelopmental disorders in part via epigenetic mechanisms, we measured OGG1- and EtOH-dependent (1) changes in epigenetic histone protein and DNA modifications, (2) altered expression of representative genes involved in epigenetic regulation and neurodevelopment, (3) involvement of candidate locus *Esr1* and (4) neurodevelopmental disorders using a battery of behavioural tests. We were particularly interested in estrogen receptors 1 and 2 (ESR1, ESR2), as one study has reported increased expression of ESR1 target genes in brains of untreated *Ogg1* −/− young mice [17], and we have found sex-dependent differences in epigenetic changes and behaviour in OGG1-deficient mice [10]. These two studies together suggest a potential role for OGG1-dependent, ESR-mediated gene regulation in brain function development. Thus, in fetal brains exposed in utero to EtOH, both gene expression and protein levels of ESR1 and ESR2 were analyzed. In addition, the association of both gene activation mark histone H3 lysine 4 trimethylation (H3K4me3) and gene repression mark histone H3 lysine 27 trimethylation (H3K27me3) [18] within various regions of the *Esr1* gene were analyzed via chromatin immunoprecipitation followed by quantitative polymerase chain reaction analysis (ChIP-qPCR) [19]. In companion studies, OGG1-deficient progeny exposed in utero to the same single low dose of EtOH were analyzed for behavioural abnormalities using tests for open field activity, social interaction, ultrasonic vocalizations and prepulse inhibition. The OGG1-dependent changes in global epigenetic marks and gene/protein expression in fetal brains, and behavioural changes, observed in both saline- and EtOH-exposed progeny suggest the involvement of epigenetic mechanisms in developmental disorders mediated by 8-oxoG and/or OGG1.

## 2. Methods

### 2.1. Animals and Diet

All animal protocols were approved by the institutional animal care committee in conformance with the guidelines established by the Canadian Council on Animal Care. *Ogg1* knockout mice on a 129SV/C57BL/6J background strain were originally generated by Klungland and coworkers [2] and were generously provided by Dr. Tomas Lindahl (Imperial Cancer Research Fund, UK) through Dr. Christi A. Walter at the University of Texas Health Science Center at San Antonio. Based on single nucleotide polymorphism analysis (SNP) (Jackson Laboratory) that identifies ~150 SNPs and covers 19 autosomes and the X chromosome, this *Ogg1* mouse strain was identified as 58% C57BL/6 and 42% 129. Mice were housed in vented plastic cages (Allentown Inc., Allentown, NJ, USA) with ground corncob bedding (Bed-O’Cobs Laboratory Animal Bedding; The Andersons Industrial Products Group, Maumee, OH, USA). Mouse cages were maintained in a room with controlled light (14 h light–10 h dark cycle) and climate (21–23 °C, with approximately 40–50% room humidity). Mice were provided with rodent chow (Harlan Labs: 2018 Harlan Teklad, Montreal, QC, Canada) and water (U.V. sterilized reverse osmosis water acidified to a pH of 3 using HCl to minimize pathogen dissemination) ad libitum. Breeder pairs were set up by placing one sexually naïve *Ogg1* +/− male with one sexually naïve *Ogg1* +/− female per cage, which were left for their entire lifetime to generate progeny of all three genotypes (+/+, +/− and −/−) within the same litter. The number of animals used in each experiment is shown in each figure in parentheses or its legend.

### 2.2. Treatment Regimen

One *Ogg1* heterozygous (+/−) knockout female mouse was mated overnight with one *Ogg1* +/− male, and the presence of vaginal plug in the morning was designated as gestation day (GD) 1. GD 17 pregnant dams were given an intraperitoneal (i.p.) injection of a single dose of 2 g/kg EtOH (25% solution (*v*/*v*) in saline) or its saline vehicle. GD 17 lies within the sensitive time window for the development of brain function in mice, and we have shown in several mouse strains that in utero exposure on this day to a range of ROS-enhancing drugs reproducibly causes neurodevelopmental disorders similar to those observed in humans [3,4,7,20]. For EtOH, the 2 g/kg dose in *Ogg1* females produces blood alcohol concentrations equivalent in humans to 4–6 drinks (based on women weighing 100–130 pounds) (Cheng et al., unpublished data), and in *Ogg1* mice causes neurodevelopmental disorders similar to those observed in human FASDs without maternal toxicity or fetal morphological abnormalities [7,21]. Fetuses were sacrificed 1, 6 or 24 h post-EtOH exposure (depending on the measure), and fetal brains were removed, snap frozen and stored at −80 °C for biochemical assessments. For behavioural tests, the dams were allowed to deliver spontaneously, and the progeny were assessed after weaning as described below. For both types of assessment, progeny were obtained from at least three litters to minimize potential litter effects.

### 2.3. DNA Extraction and Determination of Ogg1 Genotype and Sex

DNA was isolated from a 1–2 mm tail snip of either fetuses or weaned progeny by heating the samples at 62 °C in 200 µL lysis solution (0.5% SDS, 0.1 M NaCl, 50 mM Tris HCl, pH 8.0, and 0.5 µM EDTA) containing 80 µg of proteinase K (8 µL of 10 mg/mL) for 3 h. After the entire tissue was dissolved, 37 µL of 8 M potassium acetate solution was added to each sample, followed by freezing samples for >15 min (or overnight) at −20 °C. Samples were spun for 5 min at 15,700× *g* in a microcentrifuge. The supernatant or aqueous top phase was transferred to a new tube and 500 µL of 100% ethanol was added to the supernatant of each sample to precipitate DNA. The samples were spun at 15,700× *g* for 3 min, and the precipitate was washed with 500 µL of 70% ethanol, followed by another centrifugation at 15,700× *g* for 2 min. The supernatant was discarded, and the samples were air dried for 15–20 min. The pellets were resuspended with 100 µL ddH_2_O and were incubated at 4 °C overnight to solubilize DNA.

DNA was genotyped for the presence or absence of *Ogg1* using a PCR-based assay. Primers (Sigma-Aldrich, St. Louis, MO, USA) used to amplify the 500-base pair (bp) band for the *Ogg1* gene were *Ogg1*-sense (5′-ACTGCATCTGCTTAATGGCC-3′) (forward primer) and *Ogg1*-antisense (5′-CGAAGGTCAGCACTGAACAG-3′) (reverse primer). Primers used to amplify the 300 bp band for the neo-cassette responsible for disruption of the *Ogg1* gene in the *Ogg1* knockout mice were neo-sense (5′-CTGAATGAACTGCAGGACGA-3′) (forward primer) and neo-antisense (5′-CTCTTCGTCCAGATCATCCT-3′) (reverse primer). PCR reaction conditions were 2 μL genomic DNA, 2 μL per sample of 10× DreamTaq Green Buffer (Thermo Scientific, Burlington, ON, Canada, Cat. EP0713), 0.4 μL per sample of 10 mM deoxyribonucleotides (dNTP) (Thermo Scientific, Cat. R0192), 0.3 μL per sample of each of the four 20 μM primers, 0.2 μL of DreamTaq DNA Polymerase (Thermo Scientific, Cat. EP0713) and 14.2 μL per sample of ddH_2_O for a final volume of 20 μL. Cycling conditions were 95 °C for 5 min and 35 cycles of 94 °C for 1 min, 55 °C for 1.5 min, 72 °C for 2 min and a final extension at 72 °C for 10 min.

### 2.4. Sex Genotyping

DNA extracted from the tail snips of fetuses (as described above) was used to assess the sex of the fetus using a published protocol [22], with ~280 bp product amplified from male DNA and ~685 bp product (along with ~480 and 660 bp products) amplified from female DNA [22]. The PCR products were separated on a 1.5% (*w*/*v*) agarose gel in 1× TAE buffer (40 mM Tris, pH 8.3–8.5, 40 mM glacial acetic acid, 1 mM EDTA, BioShop, Burlington, ON, Canada) and 8 μg ethidium bromide. The agarose gel was run at 130 V for 40 min and then visualized and photographed under an ultraviolet light.

### 2.5. Histone Modifications

Histones were isolated from fetal brain samples using a protocol adapted from Kim and Shukla 2006 [23]. Briefly, fetal brains were homogenized and nuclei were isolated by sucrose density gradient centrifugation. Histone proteins were then acid-extracted from the nuclei, and about 5 μg of protein was loaded onto an SDS-PAGE gel, which was transferred to a nitrocellulose membrane for the assessment of histone acetylation and methylation changes by western blot. Activation modifications include acetylation of histone H3 at lysine 9 (H3K9ac, Cat. 06-942, Millipore Sigma, Burlington, MA, USA) and trimethylation of histone H3 at lysine 4 (H3K4me3, Cat. ab8580, Abcam, Cambridge, UK), and repressive modifications include trimethylation of histone H3 at lysine 9 and lysine 27 (H3K9me3, Cat. 07-442, and H3K27me3, Cat. 07-449, Millipore Sigma, respectively).

### 2.6. 5-Methylcytosine (5-mC) and 5-Hydroxymethylcytosine (5-hmC) Levels in GD 17 Fetal Brains

DNA from the fetal brains was extracted using a DNA extraction kit (FitAmp General Tissue Section DNA Isolation Kit, Cat. P-1003, EpiGentek, Farmingdale, NY, USA), with minor modifications. Briefly, brains were homogenized using 2 mL of lysing buffer containing 320 mM sucrose, 5 mM MgCl_2_, 10 mM Tris, 0.1 mM deferoxamine and 1% Triton X-100, pH 7.5. The entire homogenate was centrifuged at 1000× *g* for 15 min at 4 °C, and DNA was extracted from the nuclear pellets following the procedure in the kit. A 100-ng aliquot of DNA was used to measure the levels of 5-mC using an ELISA-based kit (Cat. P-1034, EpiGentek), while 150 ng of DNA was used to measure the 5-hmC levels using an ELISA-based kit (Cat. P-1036, EpiGentek). 5-mC and 5-hmC levels were measured in the same samples. The levels were quantified as suggested in the kit.

### 2.7. Gene Expression via Reverse Transcriptase Followed by Quantitative Polymerase Chain Reaction (RT-qPCR)

A published protocol was adapted [24] with minor modifications. Fetal brains were homogenized using 1 mL of TRIzol™ LS Reagent (Cat. 10296-010, Invitrogen, Waltham, MA, USA) and incubated at room temperature for 5 min, followed by addition of 200 μL of chloroform and incubation at room temperature for 15 min. Samples were then centrifuged at 12,000× *g* for 15 min at 4 °C. In a new tube, isopropyl alcohol was added in a 1:1 ratio of isopropyl alcohol:RNA. Samples were incubated at room temperature for 10 min followed by centrifugation at 12,000× *g* for 10 min at 4 °C. The pellet was washed with 1 mL of 75% ethanol (in diethylpyrocarbonate (DEPC) water) followed by centrifugation at 7600× *g* for 5 min at 4 °C. The pellet was dissolved in 70 μL of DEPC water and its concentration was measured to be between 500 and 1000 ng/μL. The purity of RNA was determined by ensuring that the A260/A280 ratio was ~1.8–2.0 for pure RNA and the A260/A230 ratio was ~2.0. Before conversion to cDNA, samples containing 2 μg of RNA were treated with DNAse (4.2 mM MgCl_2_, 1.28 U of DNAse I, total volume 20 μL) at 37 °C for 30 min, followed by inactivation of enzyme at 75 °C for 10 min. RNA was then converted to cDNA using a High-Capacity cDNA Reverse Transcription Kit (Cat. 4368814, ThermoFisher Scientific, Waltham, MA, USA) and a 40 μL reaction was run under the following conditions: 25 °C for 10 min, 37 °C for 120 min and 85 °C for 5 min. Samples were then diluted to 10 ng/μL. About 12.5 ng of cDNA was used to run the RT-qPCR reaction. See Supplementary Figure S8 for primer information.

### 2.8. Protein Levels

Fetal brains were homogenized using radioimmunoprecipitation assay (RIPA) buffer (150 mM NaCl, 1.0% Triton X-100, 0.5% sodium deoxycholate, 0.1% SDS and 50 mM Tris, pH 8.0) containing protease inhibitor cocktail (Roche, Basel, Switzerland, Cat. No: 11836170001). The protein concentrations of the supernatants were determined with bicinchoninic acid (BCA) assay. About 75 μg of protein homogenate was loaded onto a 12.5% SDS PAGE gel. The gel was run at constant 150 V for 1 h in electrophoresis buffer (25 mM Tris base, 192 mM glycine and 0.1% sodium dodecyl sulfate, pH 8.3). The protein was transferred onto a nitrocellulose membrane using 1× Transfer Buffer (25 mM Tris base, 192 mM glycine and 20% methanol) at 100 V for 1 h 30 min. Membranes were then washed three times in Tris-buffered saline, 0.1% Tween-20 (TBST), followed by blocking using 5% non-fat milk blocking solution in TBST for 1 h at room temperature. Membranes were then washed again three times with TBST. The membranes were cut into three pieces to be able to incubate the blots with estrogen receptor 1 (1:2000, Millipore Sigma, Cat. 06-935) and estrogen receptor 2 (1:2000, Abcam, Cat. ab3576) in 5% TBST-milk and Gapdh (1:35,000, Millipore SIGMA, Cat. G9295) in 3% bovine serum albumin in TBST overnight in a cold room. The next day, membranes were incubated with secondary antibody for both ESR1 and ESR2 (Goat anti-rabbit horseradish peroxidase, 1:30,000, Millipore Sigma, Cat. A0505) in TBST for 1 h at room temperature. All membranes (including Gapdh) were then washed three times for 5 min each with TBST, followed by a 10-min single wash with TBST. Membranes were then incubated with enhanced chemiluminescence stain (Pierce ECL Plus Western Blotting Substrate, Thermo Scientific, Burlington, ON, Canada, Cat. 32132) for 5 min and analyzed with a FluorChem8800 imager.

### 2.9. Chromatin Immunoprecipitation and qPCR (ChIP-qPCR)

Chromatin was immunoprecipitated as previously described [19]. Briefly, fetal brains were homogenized in 1% formaldehyde in phosphate buffered saline (PBS) for 10 min. Cross-linking was quenched by adding 125 mM glycine for 5 min at room temperature, and the nuclear pellet was washed with PBS, pelleted and resuspended in 800 μL of lysis buffer (20 mM Tris.HCl, pH 8.0, 1% Triton X-100, 150 mM NaCl, 0.1% *w*/*v* SDS, 2 mM EDTA) containing protease inhibitors (Roche, Cat. 11836170001) and sonicated at high intensity in 30 s on, 30 s off cycles for 15 min twice using a Bioruptor (model UCD-200, Diagenode, Denville, NJ, USA). An aliquot of sonicated chromatin was set aside to represent input fraction and to verify for sonication efficiency and stored at −80 °C. The sonicated chromatin was incubated with 30 μL Protein A agarose/salmon sperm DNA slurry (50% slurry, Cat. 16-157, Sigma Millipore), under gentle rotation on a tube rotator for 1 h at 4 °C, after which the sample was centrifuged to separate the beads. The supernatant was transferred to a new low DNA-binding tube (150 μL/immunoprecipitation). 4 μg antibody and 20 μL BSA was added to each immunoprecipitation tube (IgG, H3K9ac, H3K27me3 and H3) and the samples were incubated overnight at 4 °C. The next morning, 30 μL Protein A agarose/salmon sperm DNA slurry was added and samples were incubated at 4 °C for 2 h. The pellets were successively washed three times for 10 min each in 1 mL of Tris.Saline.EDTA (TSE I) buffer (20 mM Tris.HCl, pH 8.0, 1% Triton X-100, 150 mM NaCl, 0.1% *w*/*v* SDS, 2 mM EDTA.Na_2_), twice in 1 mL of TSE II (20 mM Tris.HCl, pH 8.0, 1% Triton X-100, 500 mM NaCl, 0.1% *w*/*v* SDS, 2 mM EDTA.Na_2_), twice in 1 mL of LiCL (20 mM Tris.HCl, pH 8.0, 1 mM EDTA.Na_2_, 250 mM LiCl, 1% *v*/*v* NP-40, 1% *w*/*v* Na-deoxycholate) and three times in 1 mL of Tris.EDTA (TE) buffer (10 mM Tris.HCl pH 8.0, 1 mM EDTA.Na_2_). Protein–DNA complexes were eluted in 110 μL elution buffer (1% SDS/TE) for 30 min, and the cross-links were reversed by overnight incubation at 65 °C (5 μL of 5 M NaCl and 1 μL of RNase A). DNA was purified using the Biobasic PCR Purification kit (Cat. BS664-250) and eluted in 40 μL TE buffer. CHIP DNA (5 μL) was amplified by PCR using primers listed in Supplementary Figure S9.

### 2.10. Behavioural Tests

**Figure 1:**
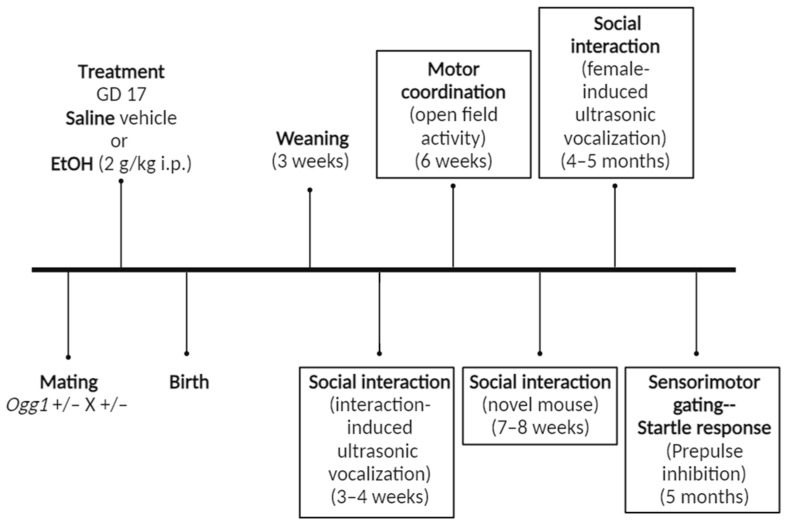
**Dosing and behavioural testing timeline**. *Ogg1* heterozygous mice were mated, and on gestational day (**GD**) 17, the dams received a single dose of EtOH (2 g/kg i.p.) or its saline vehicle. The pups were delivered spontaneously, weaned after 3 weeks and subjected to a series of behavioural tests including interaction-induced ultrasonic vocalization (USV) at 3–4 weeks of age, open field activity at 6 weeks of age, social interaction with a novel mouse at 7–8 weeks of age, female-induced USV at 4–5 months of age and prepulse inhibition at approximately 5 months of age.

After weaning at 3 weeks of age, pups were handled for three consecutive days to accustom them to human exposure and reduce stress and anxiety. The following behavioural studies were completed in a single mouse cohort at the specified times: interaction-induced ultrasonic vocalization (USV) (3–4 weeks), open field activity (6 weeks), social interaction (7–8 weeks old), female-induced USV (4–5 months) and prepulse inhibition (~5 months) (Figure 1). At least three litters were tested for each treatment group, and the number of mice tested is indicated in parentheses.

### 2.11. Interaction-Induced Ultrasonic Vocalizations (USV)

Interaction-induced USV was performed to assess social interactive behaviour at 3–4 weeks of age using lighting conditions of ~15–20 lux to minimize anxiety in the mice [25,26,27]. The test was performed by placing two mice in an empty mouse cage for 5 min with a microphone placed directly above the centre. The number of USVs emitted were recorded and manually counted, with USVs defined as vocalizations between 50 and 100 kHz. Mice were matched by age, sex, *Ogg1* genotype and treatment group.

### 2.12. Open Field Activity

Locomotor activity and anxiety were measured using an open field activity box at ~130 lux for 1 h at 6 weeks of age [20,28,29,30]. During the test, the mouse was placed in a 16-inch by 16-inch arena with sensory infrared beams that span the whole area. Breaks in these beams detect movement. The mouse was allowed to explore the arena for 1 h. Various activity parameters were recorded and analyzed.

### 2.13. Social Interaction Test

Alterations in social behaviour were assessed at 7–8 weeks of age at ~6–8 lux [25,26]. The test involved placing a mouse in a white walled arena of 62 × 40.5 × 23 cm with a novel social mouse placed inside one of the two inverted wire cups (defined as the social zone), with the other cup placed empty (defined as the nonsocial zone). The test mouse was monitored for 10 min using video tracking software. An *Ogg1* wild-type untreated mouse was used as a “novel mouse”, was age and sex-matched and was used up to about four times throughout the experiment. The novel mouse was placed first in the arena and was allowed to habituate for 2 min prior to the addition of the test mouse. A 20 × 20 cm zone was defined around each of the inverted wire cup leaving a 5 cm gap in which the whole body of the test mouse must enter to interact with the novel mouse.

### 2.14. Female-Induced Ultrasonic Vocalization (USV)

This test was performed to analyze female-induced USV in male mice [25,26]. The test was performed at ~15–20 lux to minimize anxiety in mice. The test was performed at 4–5 months of age by placing one male and one female mouse together for 5 min in an empty mouse cage with a microphone placed directly above the centre. Mice were matched by age, *Ogg1* genotype and treatment group.

### 2.15. Prepulse Inhibition

A prepulse inhibition test was performed to measure abnormalities in sensorimotor gating or startle response in OGG1 mice (~5 months old) [31]. The prepulse inhibition test was conducted using SR-LAB equipment and SR-LAB software from San Diego Instruments. Accelerometers were calibrated to 700  ±  5 mV and output voltages were amplified and analyzed for voltage changes using SR Analysis (San Diego Instruments, San Diego, CA, USA). Each mouse was tested for 30 min. The background white noise was maintained at 65 dB, and each mouse was subjected to 80 randomized trials of pulse alone (100 dB above background), prepulse alone (4, 8 or 16 dB above background), prepulse plus pulse and no pulse, with five pulse-alone trials performed at the start and end of the 80 trials. Time intervals between trials were randomized from 5 s to 20 s, with a delay of 100 ms between the prepulse and the pulse. Prepulse inhibition was thus measured as a decrease in the amplitude of startle response to a 100 dB (above background) acoustic startle pulse following each prepulse (4, 8 and 16 dB above background).

## 3. Results

### 3.1. Altered Histone and DNA Modifications in Fetal Brains of Ogg1 −/− Mice Exposed In Utero to EtOH

**Figure 2:**
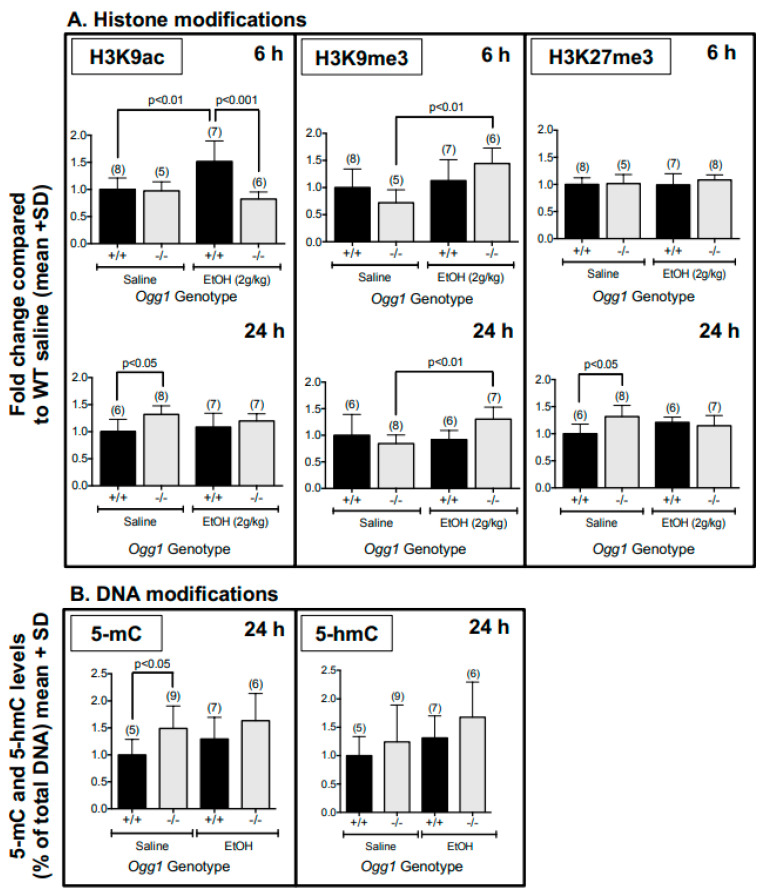
**Altered histone and DNA modifications in fetal brains of *Ogg1* −/− mice exposed in utero to EtOH.** GD 17 fetal brains exposed in utero to a single dose of EtOH (2 g/kg i.p.) or its saline vehicle were extracted 1, 6 and 24 h later from *Ogg1* +/+ and −/− littermates and were assessed for histone and DNA modifications. Fetal brains from at least three litters were used to minimize potential litter effects, and the number of fetal brains for each group is shown in parentheses. (**A**). The following histone modifications were analyzed: H3K9ac (activation mark), H3K9me3 and H3K27me3 (repressive marks). See Supplementary Figure S1 for 1 h results for H3K9ac and H3K9me3 (no differences observed) and for 6 and 24 h results for H3K4me3 (activation mark, no differences observed). (**B**). Increase in 5-methylcytosine (5-mC) levels in saline-exposed *Ogg1* −/− vs. +/+ fetal brains at 24 h, with a similar trend in EtOH-exposed fetal brains. The same DNA sample was used for both 5-mC and 5-hmC measurements. The significance of differences was determined by two-way ANOVA and a post hoc Tukey’s test. Abbreviations: 5-mC: 5-methylcytosine; 5-hmC: 5-hydroxymethylcytosine; H3K4me3: histone 3 lysine 9 trimethylation; H3K9ac: histone 3 lysine 9 acetylation; H3K9me3: histone 3 lysine 9 trimethylation; H3K27me3: histone 3 lysine 27 trimethylation.

Global histone protein activation marks (H3K9ac and H3K4me3) and repressive marks (H3K9me3 and H3K27me3) were assessed via western blot in fetal brains exposed in utero to saline or EtOH (Figure 2A, Supplementary Figure S1). EtOH exposure enhanced histone acetylation (H3K9ac) levels at 6 h in *Ogg1 +*/*+* (*p* < 0.01) but not −/− fetal brains, which had returned to baseline at 24 h. However, at 24 h, H3K9ac levels were elevated in saline-exposed −/− vs. +/+ (*p* < 0.05) but not EtOH-exposed *Ogg1* −/− vs. +/+ brains. H3K9me3 levels were increased by EtOH exposure in −/− but not +/+ brains at both 6 and 24 h (*p* < 0.01), whereas H3K27me3 levels were increased in only saline-exposed −/− vs. +/+ brains at only 24 h (*p* < 0.05).

For DNA modifications (Figure 2B), at 24 h, an OGG1-dependent increase in 5-mC levels was observed only in saline-exposed −/− vs. +/+ brains (*p* < 0.05), with no effect of EtOH observed. No differences were observed in 5-mC and 5-hmC levels at 1 or 6 h, and there were no OGG1- or EtOH-dependent changes in 5-hmC levels. The significance for both histone and DNA modifications is based on two-way ANOVA and a post hoc Tukey’s test.

### 3.2. Altered Gene Expression in OGG1 Fetal Brains Exposed In Utero to EtOH

**Figure 3:**
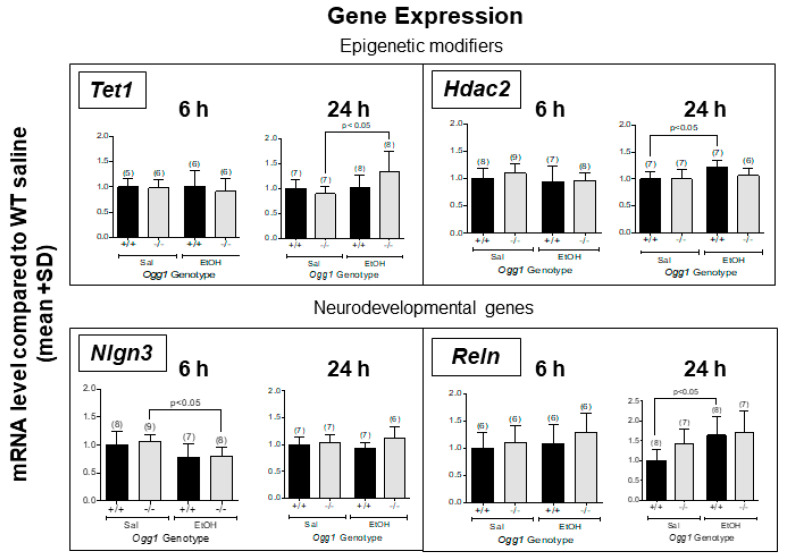
**Altered gene expression in *Ogg1* −/− fetal brains exposed in utero to EtOH.** GD 17 fetal brains exposed in utero to a single dose of EtOH (2 g/kg i.p.) or its saline vehicle were extracted 6 and 24 h later from *Ogg1* +/+ and −/− littermates. Fetal brains from at least three litters were used to minimize potential litter effects, and the number of fetal brains for each group is shown in parentheses. Fetal brains were homogenized, RNA was extracted using the TRIzol method and mRNA expression levels were measured via RT-qPCR. *Gapdh* was used as a control. Expression levels of various learning and memory candidate genes were measured (see Supplementary Figure S2). Above are the results for OGG1- and EtOH-dependent differences in mRNA levels in fetal brains. The significance of differences was determined by two-way ANOVA and a post hoc Tukey’s test. Abbreviations: *Hdac2:* histone deacetylase 2; *Nlgn3*: neuroligin 3; *Reln:* Reelin; *Tet1:* Ten-eleven translocation methylcytosine dioxygenase 1.

Expression levels of representative genes involved in learning and memory were measured via RT-qPCR. In *Ogg1* −/− fetal brains, EtOH increased *Tet1* expression (24 h, *p* < 0.05) and decreased *Nlgn3* expression (6 h, *p* < 0.05) compared to saline (Figure 3). In contrast, in *Ogg1* +/+ fetal brains, EtOH increased *Hdac2* and *Reln* expression (*p* < 0.05) compared to saline, neither of which were altered in *Ogg1* −/− progeny. See Supplementary Figure S2 for a list of other genes with no OGG1- or EtOH-dependent differences. The significance is based on two-way ANOVA and a post hoc Tukey’s test.

### 3.3. OGG1-and Sex-Dependent Differences in Esr1 and Esr2 mRNA Levels and Their Ratios in Fetal Brains Exposed In Utero to EtOH

**Figure 4:**
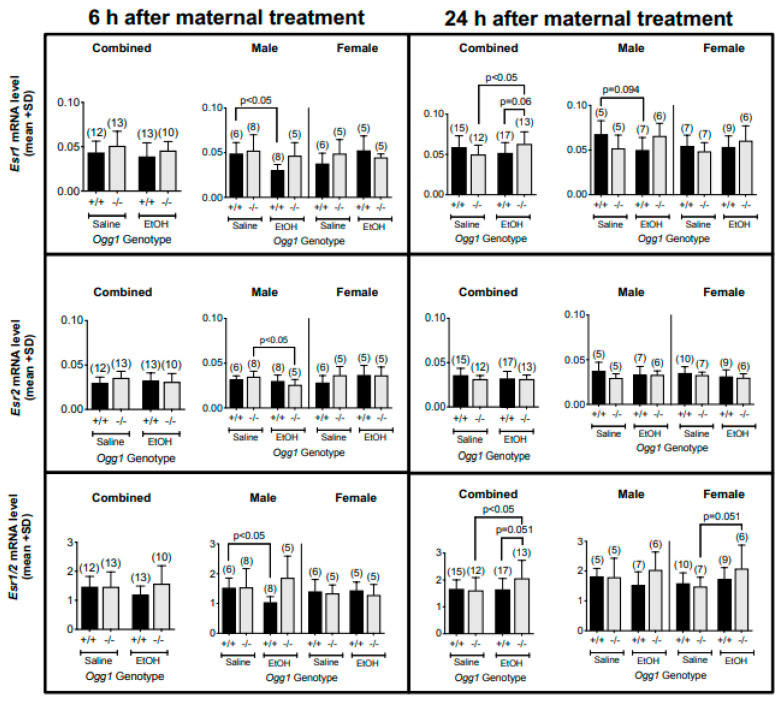
**OGG1- and sex-dependent differences in *Esr1* and *Esr2* mRNA levels and their ratios in fetal brains exposed in utero to EtOH**. GD 17 fetal brains exposed in utero to a single dose of EtOH (2 g/kg i.p.) or its saline vehicle were extracted 6 and 24 h later from *Ogg1* +/+ and −/− littermates. Fetal brains from at least three litters were used to minimize potential litter effects, and the number of fetal brains for each group is shown in parentheses. Fetal brains were homogenized, RNA was extracted using the TRIzol method and mRNA expression levels were measured via RT-qPCR. Gapdh was used as a control. Above are the results for *Esr1* and *Esr2* mRNA levels as well as their ratios in fetal brains. The significance of differences was determined by two-way ANOVA and a post hoc Tukey’s test. Abbreviations: *Esr1*: estrogen receptor 1; *Esr2*: estrogen receptor 2.

Since one study has reported increased expression of ESR1-regulated genes (i.e., ESR1 target genes) in brains of untreated *Ogg1* −/− young mice [17], we analyzed the *Esr1* gene expression separated by sex (Figure 4). In the combined sex data, at 6 h, no differences were observed in expression levels for *Esr1*, *Esr2* or the *Esr1*/*2* ratio, whereas at 24 h, EtOH-exposed *Ogg1* −/− fetal brains showed a small but significant increase in *Esr1* expression (*p* < 0.05) in comparison to saline-exposed *Ogg1* −/− fetal brains, and among EtOH-exposed progeny, *Esr1* expression was marginally increased in *Ogg1* −/− fetal brains (*p* = 0.06) compared to *Ogg1* +/+ littermates. When the data were separated by sex, *Esr1* expression at 6 h was decreased in EtOH- vs. saline-exposed *Ogg1* +/+ male fetal brains (*p* < 0.05), but not in *Ogg1* −/− male brains nor in female fetal brains. At 24 h, although not significant, a similar trend for decreased mRNA was seen in male fetal brains. *Esr2* gene expression at 6 h was decreased in EtOH vs. saline-exposed *Ogg1* −/− (*p* < 0.05) but not in +/+ male brains nor in female fetal brains. No significant differences were observed in *Esr2* levels at 24 h. When analyzed for *Esr1*/*2* ratio, there was a decrease in its ratio at 6 h in EtOH-exposed *Ogg1* +/+ male fetal brains but not −/− fetal brains (*p* < 0.05), with no differences observed in the females. The significance is based on two-way ANOVA and a post hoc Tukey’s test.

### 3.4. OGG1-and Sex-Dependent Differences in ESR1 and ESR2 Protein Levels and Their Ratios in Fetal Brains Exposed In Utero to EtOH

**Figure 5:**
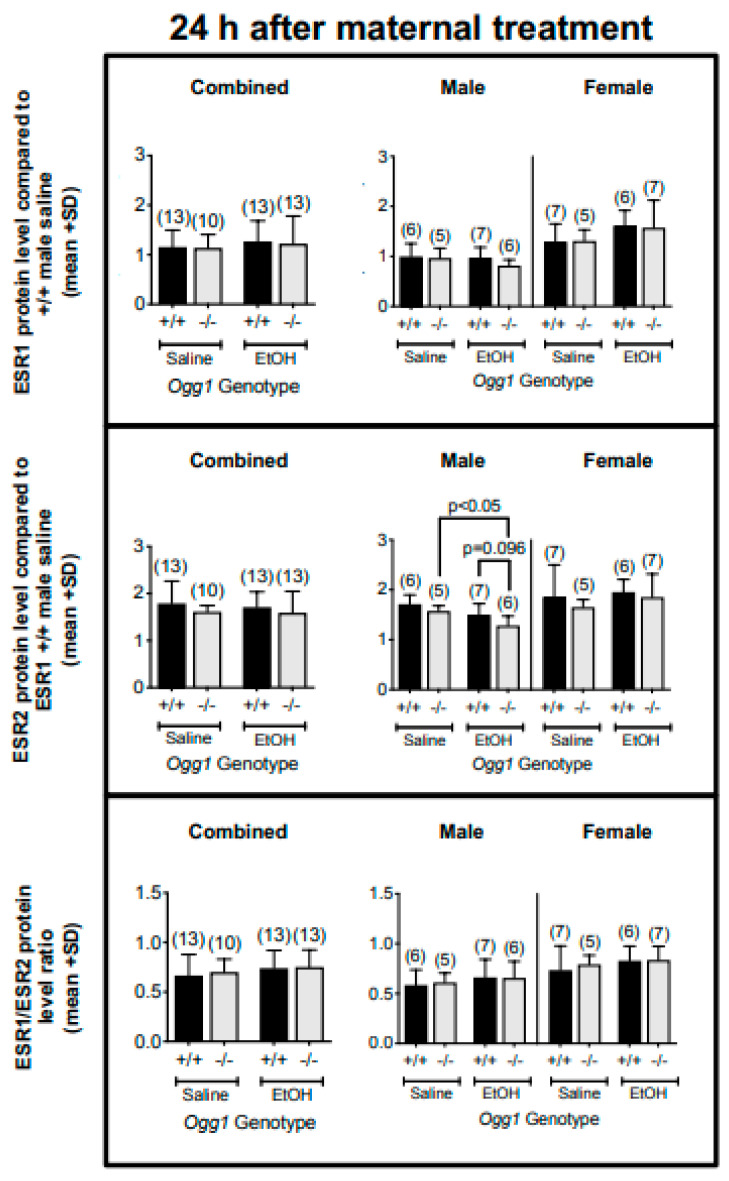
**OGG1- and sex-dependent differences in ESR1 and ESR2 protein levels and their ratios in fetal brains exposed in utero to EtOH**. GD 17 fetal brains exposed in utero to a single dose of EtOH (2 g/kg i.p.) or its saline vehicle were extracted 6 and 24 h later from *Ogg1* +/+ and −/− littermates. Fetal brains from at least three litters were used to minimize potential litter effects, and the number of fetal brains for each group is shown in parentheses. Fetal brains were homogenized, and protein levels were quantified via western blot. GAPDH was used as a loading control. Above are the results for ESR1 and ESR2 proteins levels as well as their ratios in fetal brains. The significance of differences for each sex was determined by two-way ANOVA and a post hoc Tukey’s test. Abbreviations: ESR1: estrogen receptor 1; ESR2: estrogen receptor 2.

Protein levels were measured at 24 h post maternal treatment (Figure 5). No differences were seen in ESR1 protein levels; however, ESR2 protein levels were decreased in EtOH- vs. saline-exposed *Ogg1* −/− male fetal brains (*p* < 0.05), but not in *Ogg1* +/+ male brains nor in female fetal brains. No differences in ESR1/ESR2 protein level ratios were seen. The significance is based on two-way ANOVA and a post hoc Tukey’s test.

### 3.5. EtOH-Mediated Increased Association of H3K27me3 with Esr1 Gene in Ogg1 +/+ but Not −/− Fetal Brains

**Figure 6:**
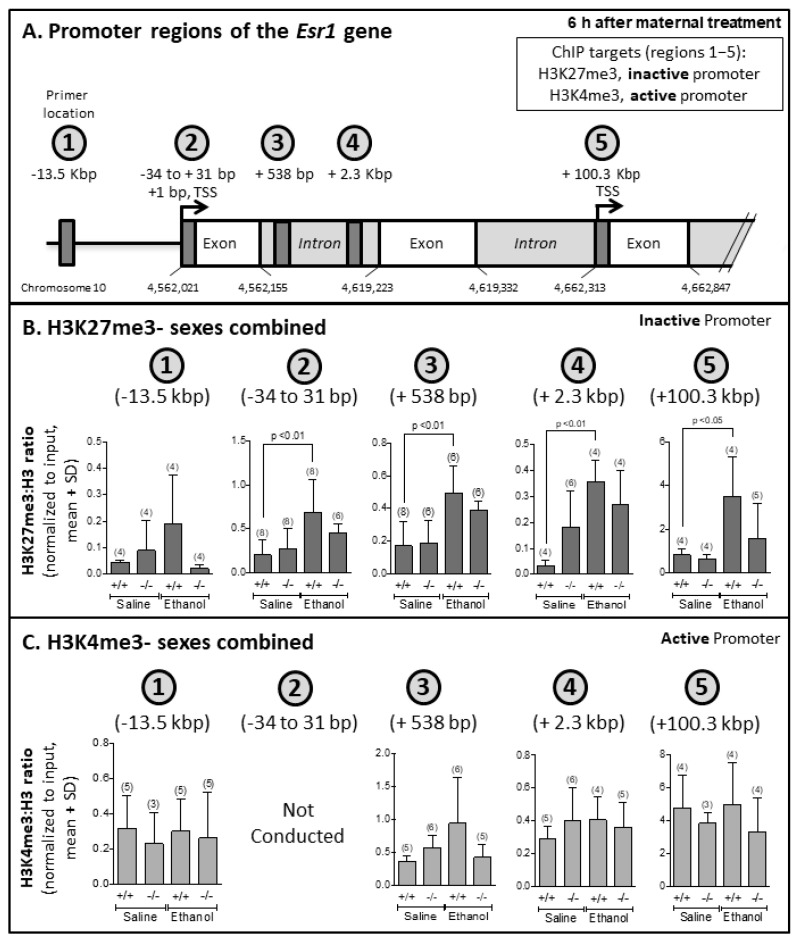
**EtOH-mediated increased association of H3K27me3 with *Esr1* gene expression in *Ogg1* +/+ but not −/− fetal brains.** GD 17 fetal brains exposed in utero to a single dose of EtOH (2 g/kg i.p.) or its saline vehicle were extracted 6 h later from *Ogg1* +/+ and −/− littermates. Fetal brains from at least three litters were used to minimize potential litter effects, and the number of fetal brains for each group is shown in parentheses. (**A**). ChIP was performed using fetal brains, and extracted DNA was used to perform quantitative PCR using five different sets of primers directed against various regions of the *Esr1* gene. The *Esr1* gene has two promoter regions, marked with arrows, which can generate four transcript variants via gene splicing. The locations of the exons and introns on chromosome 10 are marked. The primer locations are relative to the first transcription start site (**TSS**). Regions 1–5 were chosen based on the Ensembl database reporting their association with activation or repressive epigenetic marks. Each of the regions was amplified after chromatin was immunoprecipitated using antibodies against H3K4me3 (active promoter) and H3K27me3 (inactive promoter) and histone H3 (control). This figure is not drawn to scale. (**B**). The association of the H3K27me3:H3 ratio was normalized to 1% input in various regions of *Esr1* of fetal brains exposed in utero to saline or EtOH. (**C**). The association of the H3K4me3:H3 ratio was normalized to 1% input in various regions of *Esr1* of fetal brains exposed in utero to saline or EtOH. The significance of differences was determined by two-way ANOVA and a post hoc Tukey’s test. See Supplementary Figure S3 for controls and Figures S4 and S5 for sex-separated data.

The association of histone modifications H3K27me3 (repression mark) and H3K4me3 (activation mark) within various regions of the *Esr1* gene were quantified via chromatin immunoprecipitation followed by quantitative PCR (Figure 6A). We chose these marks based on the Ensembl database, which showed a high association of activation mark H4K3me3 around the *Esr1* promoter, and to complement this activation mark, we chose to also measure the repression mark H3K27me3. At 6 h, there was an increased association of H3K27me3 in EtOH- vs. saline-exposed *Ogg1* +/+ but not −/− progeny at various regions of the *Esr1* gene (relative to transcription start site (+ 1 bp): −34 to 31 bp (*p* < 0.01), + 538 bp (*p* < 0.01), + 2.3 kbp (*p* < 0.01) and + 100.3 kbp (*p* < 0.05)) (Figure 6B). No OGG1- or EtOH-dependent differences in H3K4me3 levels within the *Esr1* gene were observed (Figure 6c). See Supplementary Figure S3 for controls and Figures S4 and S5 for sex-separated data with low n. The significance is based on two-way ANOVA and a post hoc Tukey’s test.

### 3.6. Increased Hyperactivity in Saline- but Not EtOH-Exposed Ogg1 −/− vs. +/+ Females and Ogg1- and Sex-Dependent Changes in Centre Time Spent in Saline- and EtOH-Exposed Progeny

**Figure 7:**
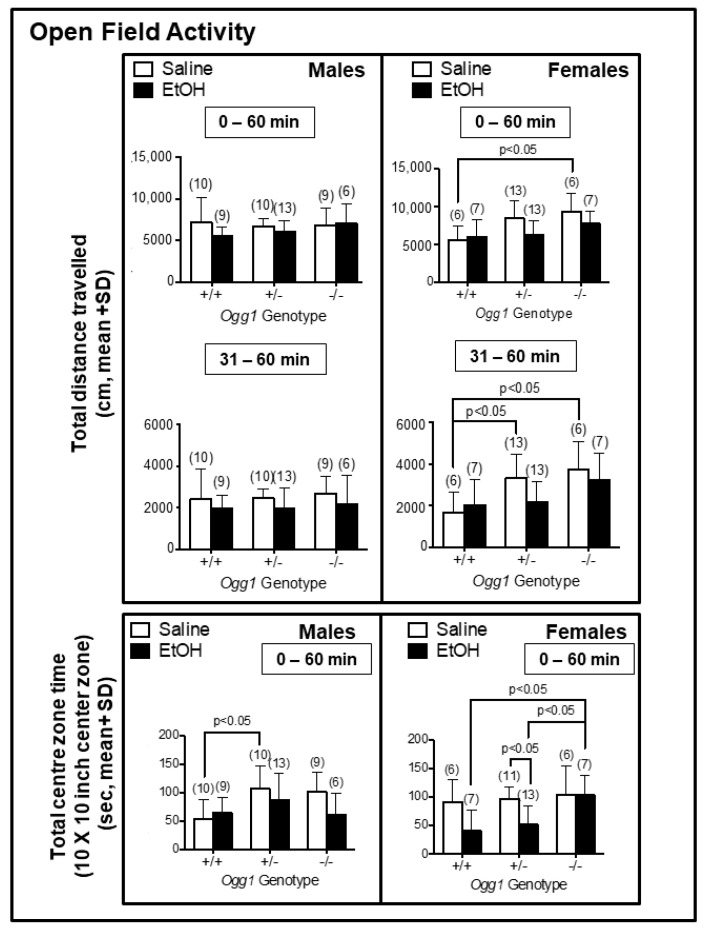
Increased hyperactivity for saline- but not EtOH-exposed *Ogg1* −/− vs. +/+ females, and decreased centre time for EtOH-exposed *Ogg1* +/+ but not −/− female mice. For all behavioural studies, pregnant dams were treated with single dose of EtOH (2 g/kg i.p.) or its saline vehicle on GD 17, as shown in Figure 1, and progeny were delivered spontaneously. Fetal brains from at least three litters were used to minimize potential litter effects, and the number of mice tested for each group is shown in parentheses. Results show total distance travelled during the entire test (1 h), total distance travelled during the last 30 min of the test and total time spent in the centre zone (10 × 10 inches). The significance of differences was determined by two-way ANOVA and a post hoc Tukey’s test. See Supplementary Figure S6a for data for the nonsocial zone.

Increased hyperactivity was observed in saline-exposed *Ogg1* −/− vs. +/+ females (*p* < 0.05) but not males when averaged over 1 h (Figure 7). When analyzed for the last half hour, there was increased hyperactivity in saline-exposed *Ogg1* −/− and +/− vs. +/+ females (*p* < 0.05). Although similar trends were seen with in utero EtOH exposure, they were not significant. Analysis of centre zone activity revealed that saline-exposed *Ogg1* +/− vs. +/+ male progeny showed greater time spent in the centre zone, but there was no *Ogg1*-dependent effect in saline-exposed females. In contrast, in utero EtOH exposure decreased the total time spent in the centre zone by *Ogg1* +/− female progeny compared to +/− saline controls (*p* < 0.05), but had no effect on males. EtOH exposure also increased total time spent in the centre zone by *Ogg1* −/− female progeny compared to both EtOH-exposed +/− and +/+ female littermates (*p* < 0.05). Interestingly, the increased time spent by EtOH-exposed *Ogg1* −/− females was similar to that observed in saline-exposed female progeny of all *Ogg1* genotypes. The significance is based on two-way ANOVA and a post hoc Tukey’s test.

### 3.7. OGG1- and Sex-Dependent Effect on Social Interaction and Interaction-Induced Ultrasonic Vocalizations but Not Startle Response

**Figure 8:**
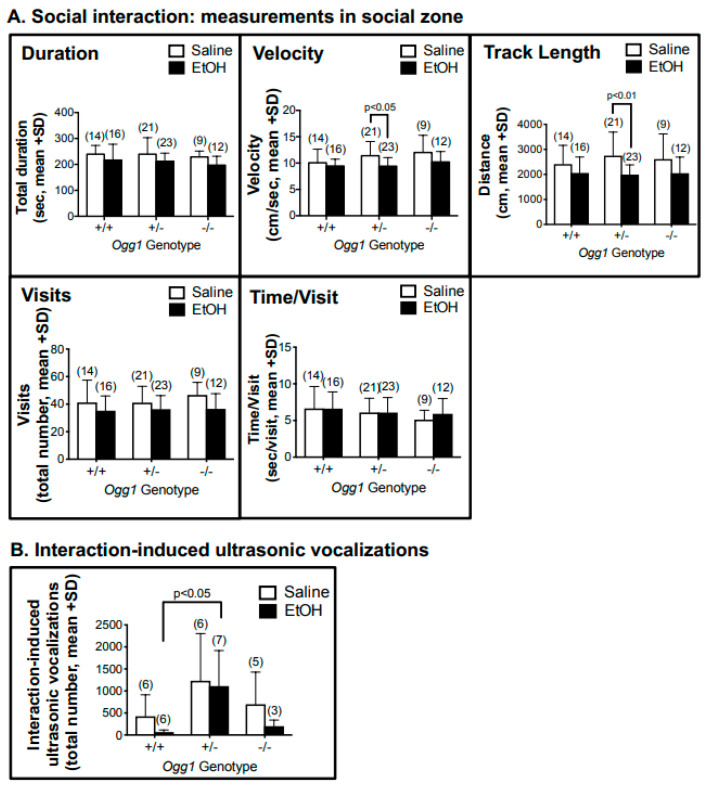
**OGG1- and sex-dependent effect on social interaction and interaction-induced ultrasonic vocalizations.** For all behavioural studies, pregnant dams were treated with a single dose of EtOH (2 g/kg i.p.) or its saline vehicle on GD 17, as described in Figure 1, and progeny were delivered spontaneously. Fetal brains from at least three litters were used to minimize potential litter effects, and the number of mice tested for each group is shown in parentheses. (Panel **A**). For social interaction, EtOH vs. saline decreased velocity and track length in *Ogg1* +/− progeny, but not in +/+ or −/− littermates, although the latter exhibited a similar non-significant trend. (Panel **B**). As with social interaction, EtOH vs. saline increased interaction-induced ultrasonic vocalizations in *Ogg1* +/− progeny, but not in +/+ or −/− littermates. The significance of differences was determined by two-way ANOVA and a post hoc Tukey’s test.

No OGG1-dependent or EtOH-dependent differences were observed in social interaction, including total duration in social zone, visits or time/visit (Figure 8A). No sex-dependent differences were observed, so the data were combined. Interestingly, EtOH- vs. saline-exposed *Ogg1* +/− progeny exhibited decreased velocity and track length in the social zone, with no differences in *Ogg1* +/+ and −/− mice (see Supplementary Figure S6a for data for the nonsocial zone).

EtOH-exposed *Ogg1* +/− progeny showed increased interaction-induced ultrasonic vocalizations (USVs) compared to EtOH-exposed *Ogg1* +/+ littermates (*p* < 0.05), with a similar trend observed in saline-exposed *Ogg1* +/− mice (Figure 8B). Unexpectedly, *Ogg1* −/− mice were similar to *Ogg1* +/+ mice (Figure 8B). No differences were observed in female-induced USVs (see Supplementary Figure S6b). The significance is based on two-way ANOVA and a post hoc Tukey’s test.

### 3.8. Prepulse Inhibition

No differences in startle response measured via prepulse inhibition were observed due to *Ogg1* genotype, sex or treatment (see Supplementary Figure S7).

## 4. Discussion

### 4.1. Overview

**Figure 9:**
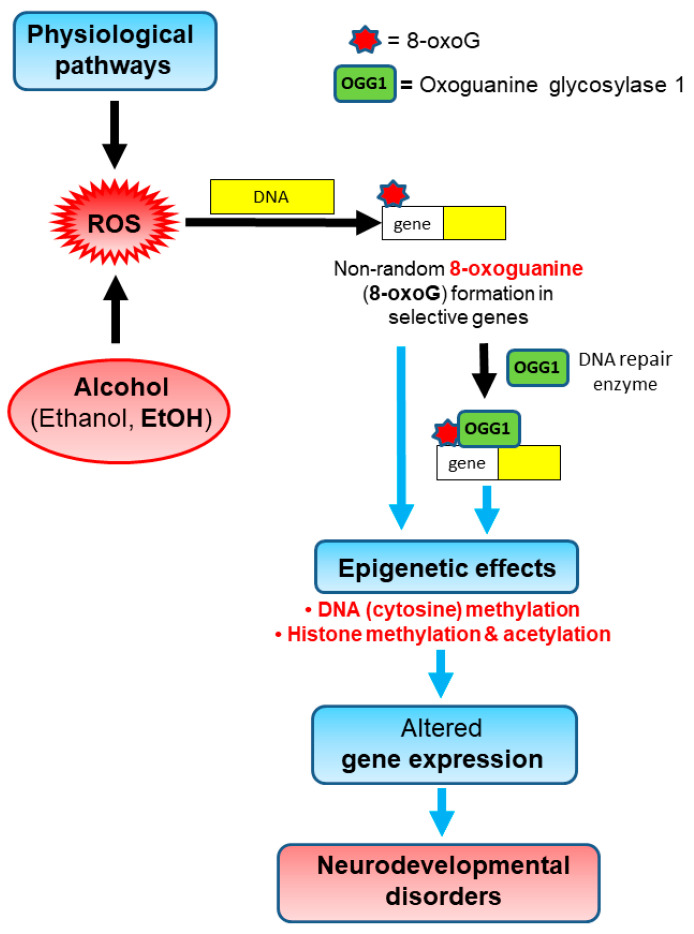
**Postulated epigenetic role of oxidative DNA damage initiated by reactive oxygen species (ROS) in neurodevelopmental disorders caused by physiological or ethanol-enhanced levels of ROS formation**. ROS, which are naturally produced in the body and essential for normal development and life, can oxidatively damage DNA, resulting in multiple types of lesions. The most prevalent DNA lesion, 8-oxoguanine (8-oxoG), is developmentally pathogenic, and is repaired by oxoguanine glycosylase 1 (OGG1). Heterozygous (+/−) and particularly homozygous (−/−) *Ogg1* knockout progeny have decreased DNA repair activity compared to their wild-type (+/+) littermates, and can accumulate oxidative DNA damage due to even physiological levels of ROS formation, leading to epigenetic changes in DNA methylation and modifications to histone proteins, exemplified by histone methylation and acetylation. These epigenetic changes can alter the expression of developmentally important genes, leading to neurodevelopmental disorders. Prenatal exposure to ROS-enhancing drugs like alcohol (ethanol, EtOH) can further enhance both the complexity and magnitude of epigenetic changes initiated by oxidative DNA damage, increasing the spectrum and severity of neurodevelopmental disorders.

OGG1- and/or EtOH-dependent changes were observed in most biochemical outcomes in whole fetal brains and postnatal behavioural disorders, which in some cases were sex-dependent, as summarized in Table 1. The OGG1-dependent biochemical changes in fetal brains exposed to saline potentially reflect the role of oxidative DNA damage in DNA repair-deficient progeny due to physiological levels of ROS formation (Figure 9). This oxidative DNA damage may contribute to developmental disorders not dependent upon xenobiotic exposures, such as some components of attention-deficit hyperactivity disorders, obsessive-compulsive disorders and autism spectrum disorders. The changes in representative epigenetic histone/DNA marks and behavioural disorders are consistent with our hypothesis (Figure 9) and provide a rationale for more comprehensive mechanistic studies to determine causal relationships. The absence of changes in gene expression with saline exposure may in part reflect the limited selection of representative genes assessed herein, and/or the use of whole fetal brains rather than brain regions or brain cell types. Also, the direction and time course of epigenetic changes and gene expression may vary within different brain regions and brain cell types, and for different genes and their associated histone proteins. Finally, although the limited behavioural tests were consistent with our hypothesis, and provided a proof of concept, a more comprehensive battery of tests would be necessary to determine the full breadth of brain functions affected by physiological levels of ROS formation in DNA repair-deficient progeny.

Prenatal exposure to the ROS-enhancing drug EtOH was similarly associated with OGG1-dependent changes in epigenetic marks in the fetal brain, but with different patterns than with saline and more extensive behavioural disorders (Table 1). In addition, unlike saline, EtOH altered the expression of all the representative genes, and a specific *Esr1* gene histone inactivation mark and ESR2 protein levels, in an OGG1-dependent fashion. In certain cases, consistent changes were not observed among epigenetic changes, estrogen receptor gene/protein levels and behaviour. These inconsistencies may have been due in part to our use of whole fetal brains rather than brain regions or specific cell types, limited sampling times, and the limited number and particular selection of representative genes. The data for prenatal EtOH exposure provide an even more compelling proof of concept consistent with our hypothesis for a role of oxidative DNA damage in epigenetic mechanisms of neurodevelopmental disorders relevant to FASD (Figure 9) and a rationale for more comprehensive mechanistic studies including whole-genome and proteomic approaches to determine causal relationships.

### 4.2. Ethanol-Initiated Alterations in Histone Modifications and Gene Expression in Fetal Brains

EtOH causes several epigenetic modifications, which have been associated with its embryopathic mechanism [14,15,16]. The effect of EtOH on histone acetylation patterns depends on the EtOH treatment paradigm, timing of exposure and brain regions examined, with varying results even within a region [16,32,33].

Herein, an EtOH-initiated increase in global H3K9ac indicated early gene activation in *Ogg1* +/+ but not −/− fetal brains, which returned to baseline at a later time point, likely due to increased HDAC2 activity, as an increase in *Hdac2* mRNA levels was observed in EtOH-exposed *Ogg1* +/+ progeny at 24 h. The initial increase in H3K9ac in EtOH-exposed *Ogg1* +/+ brains may play a role in regulating gene transcription to maintain the redox balance, as previously proposed [8]. Under oxidative stress conditions, recognition of 8-oxoG by OGG1 results in the oxidation of a cysteine sulfhydryl group in OGG1, which in its oxidized state can bind but not repair 8-oxoG. Subsequent assembly of transcriptional machinery and regulation of gene transcription restores the redox state of the cell. This in turn results in reduced OGG1, thereby excising 8-oxoG, suggesting a role for OGG1 in gene transcription regulation [8]. This is consistent with the observed increase in H3K9ac in EtOH- vs. saline-exposed *Ogg1 +*/*+* but not *Ogg1* −/− fetal brains. EtOH exposure also resulted in an increase in H3K9me3, indicating gene suppression, in *Ogg1* −/− but not +/+ fetal brains, suggesting OGG1 dependence. These results suggest that EtOH causes OGG1-dependent alterations in histone marks that alter gene expression. Although the above levels were measured globally, it is possible that EtOH may increase or decrease these marks in an *Ogg1* gene-dependent manner, as 8-oxoG formation has recently been shown to be gene-selective [34,35].

EtOH-initiated, OGG1-dependent alterations in gene expression were found for only a few of representative genes tested (see Supplementary Figure S2). Aside from the limited number of representative genes examined herein, this may be in part because the gene expression changes in our study were measured earlier in life (i.e., in the fetal brain), whereas some gene expression changes may not be seen until later in life (postnatally), exemplified by OGG1-dependent differences in gene expression in *Ogg1* −/− vs. +/+ untreated adult hippocampi around 6 months of age [17]. In addition, the use of a single low dose of EtOH may result in negligible or minor differences in gene expression, and could thus limit the quantitative discrimination of small differences in mRNA level observed via RT-qPCR when only one housekeeping gene is used as a control [36], as was the case herein (i.e., Gapdh).

In this study, the EtOH-mediated increase in Ten-eleven translocation methylcytosine dioxygenase 1 (*Tet1*) gene expression in *Ogg1* −/− but not *Ogg1* +/+ fetal brains may have contributed to altered methylation levels at the later time point, as TET proteins are involved in active demethylation of 5-mC to cytosine [37]. Reelin (RELN), along with its essential adapter protein, Disabled-1 (Dab1), regulates neuronal plasticity and subsequently memory formation [38]. A deficiency in Reelin can result in behavioural dysfunction and may contribute to schizophrenia [39] or autism [40]. One study suggests that exposure to EtOH initially activates but later silences the Reelin–Dab1 signaling pathway via brief activation and subsequently inactivation of Src-family kinases both in vitro and in vivo [41]. Interestingly, we saw an EtOH-dependent increase in *Reln* mRNA levels in *Ogg1* +/+ but not *Ogg1* −/− fetal brains, possibly because baseline *Reln* levels seemed to be already higher in saline-exposed *Ogg1* −/− fetal brains, resulting in no further increase with EtOH exposure. EtOH appeared to transiently decrease neuroligin 3 (*Nlgn3*) regardless of *Ogg1* genotype, although the decrease was significant only in *Ogg1* −/− fetal brains. Neuroligin-3 (NLGN3) is an X-linked neuronal cell surface protein and a postsynaptic cell adhesion molecule involved in the formation and remodeling of the central nervous systems [42]. One study reported decreased methylation levels in the *Nlgn3* gene with EtOH exposure in embryo culture without altering its expression [43]. A gain of function of *Nlgn3* resulting in inhibition of synaptic transmission is associated with a model for autism spectrum disorders [44].

### 4.3. Alterations in Histone Modifications, DNA Methylation and Gene Expression in Fetal Brains Exposed to Saline

For some measures, OGG1-dependent changes were seen only in saline-exposed *Ogg1* −/− fetal brains. The increase in repression marks H3K27me3 and 5-mC in saline-exposed *Ogg1* −/− vs. +/+ fetal brains at 24 h suggests that OGG1 can regulate gene methylation levels. A role for OGG1 in oxidative stress-induced DNA demethylation via recruiting TET1 to the 8-oxoG lesion has been reported [45]. This is consistent with our results showing an increase in repression marks only in *Ogg1* −/− fetal brains. This suggests OGG1 may be important in maintaining normal levels of repressive marks by facilitating the demethylation process. In addition, there was also an OGG1-dependent increase in H3K9ac in saline-exposed *Ogg1* −/− vs. +/+ brains at 24 h, which could be involved in the activation of genes facilitating recovery after the stress of a single injection and/or suggest a role for OGG1 in regulating histone acetylation marks similar to its regulation of DNA demethylation.

### 4.4. OGG1- and Sex-Dependent Differences in mRNA and Protein Levels of ESR1 and ESR2 and Their Ratios in Fetal Brains, and the Association of H3K27me3 and H3K4me3 with the Esr1 Gene

Previous evidence suggests that OGG1 may be involved in estrogen receptor (ESR)-mediated gene transcription [17,46]. For example, during estrogen-bound ESR-mediated gene transcription, a lysine-specific demethylase 1 (LSD1) demethylates histone H3 lysine 9 dimethylation marks (H3K9me2), and in the process produces localized ROS and 8-oxoG formation in discrete foci including at the promoters of various ESR1 target genes. Localized 8-oxoG recruits OGG1, which nicks the DNA, resulting in conformational changes of chromatin necessary for estrogen-dependent transcription [46].

ESRs are nuclear hormone receptors and transcription factors [47] that serve various functions, including playing an important neuroprotective role in memory formation and maintenance [47]. ESR1 inhibition is important in the formation of long-term memory in the mouse hippocampus [48], and a role for OGG1 in repressing ESR1 to modulate learning and memory formation has been postulated because hippocampal regions of *Ogg1* KO mice exhibited upregulation of *Esr1* target genes [17]. We previously saw OGG1- and sex-dependent enhanced DNA strand breaks, decreased DNA methylation levels and behaviour deficits including alterations in spatial and recognition memory in untreated OGG1-deficient young mice (~2–3 months) [10]. The presence of sex-dependent differences in young OGG1-deficient mice suggests that estrogen may play an important role in regulating gene expression in an OGG1-dependent manner, particularly during development, so *Esr1* and *Esr2* mRNA levels and protein levels were quantified in fetal brains.

Only limited OGG1- and treatment-dependent differences were seen in *Esr1* gene expression. A marginal increase in *Esr1* expression in EtOH-exposed *Ogg1* −/− vs. +/+ fetal brain (sexes combined) was associated with a consistent trend for a decrease in gene-specific H3K27me3 marks measured via ChIP-qPCR in EtOH-exposed *Ogg1* −/− vs. +/+ fetal brains across various regions of the *Esr1* gene **(**Figure 6), suggesting that EtOH can alter the ESR1 signaling pathway in an OGG1- and sex-dependent manner. Furthermore, this increase in *Esr1* expression in EtOH-exposed *Ogg1* −/− vs. +/+ fetal brains seemed to be due to an EtOH-dependent decrease in *Esr1* expression and the *Esr1*/*Esr2* ratio in EtOH- vs. saline-exposed *Ogg1* +/+ but not *Ogg1* −/− male fetal brains. This was reflected by ChIP-qPCR results, which showed an increased association of H3K27me3 (repression mark) in EtOH- vs. saline-exposed *Ogg1* +/+ but not *Ogg1* −/− brains. Analysis of ChIP-qPCR data by sex suggested that, although a trend for EtOH-mediated increase in H3K27me3 in *Ogg1* +/+ fetal brains was observed in both males and females (Supplementary Figure S4), a similar trend for an EtOH-mediated increase in H3K4me3 (activation mark) in *Ogg1* +/+ brains seemed to occur only in females (Supplementary Figure S5). An increase in both H3K27me3 (repressor) and H3K4me3 (activator) in females may cancel each other, resulting in no difference in *Esr1* gene expression in EtOH-exposed *Ogg1* +/+ females, whereas an increase only in H3K27me3 but not H3K4me3 in males may cause decreased *Esr1* gene expression in EtOH- vs. saline-exposed *Ogg1* +/+ males.

A decrease in *Esr1* gene expression in EtOH- vs. saline-exposed *Ogg1* +/+ but not −/− progeny suggests a role for OGG1 and possibly 8-oxoG in regulating *Esr1* gene expression. Mapping of 8-oxoG in the genome of *Ogg1* −/− vs. +/+ mouse embryonic fibroblasts revealed a three-fold enrichment of 8-oxoG peaks in the *Esr1* gene in *Ogg1* +/+ but not *Ogg1* −/− cells [34]. A decrease in 8-oxoG peaks in the *Esr1* gene in *Ogg1* −/− fetal brains may alter *Esr1* gene expression in *Ogg1* −/− progeny, as the presence of 8-oxoG may either increase or decrease gene expression depending on the site oxidized [8]. Interestingly, the EtOH-mediated increase in association of H3K27me3 marks with various regions of the *Esr1* gene and decreased *Esr1* gene expression in *Ogg1* +/+ male mice but not −/− male mice further suggests a role for EtOH in altering the ESR1 signaling pathway in both an OGG1- and sex-dependent manner. We hypothesize that EtOH, being a ROS inducer, may further increase 8-oxoG levels in the *Esr1* gene in both *Ogg1* +/+ and −/− fetal brains. However, in *Ogg1* +/+ brains, 8-oxoG may be recognized by OGG1, resulting in the recruitment of epigenetic modifiers and hence increased H3K27me3 levels and gene repression, whereas in *Ogg1* −/− mice lacking OGG1, 8-oxoG cannot be recognized by OGG1 and thus the repressive complex may not be recruited as efficiently as in *Ogg1* +/+ brains, resulting in no changes in H3K27me3 levels or gene repression. In a related example, OGG1 can recruit chromodomain helicase DNA-binding protein 4 (CHD4) to interact with 8-oxoG sites in genes, and CHD4 recruits repressive epigenetic modifiers complexes including DNA methyltransferases and histone H3K27 methyltransferases (such as EZH2 and G9a) to DNA damage sites, resulting in silencing of genes [49]. Thus, EtOH-enhanced ROS and 8-oxoG levels specifically in the *Esr1* gene may act similarly to repress *Esr1* gene transcription mediated by OGG1, as previously postulated [17].

The absence of differences in ESR1 protein levels may have been due in part to limited differences in *Esr1* mRNA levels and/or the time point analyzed. The protein levels were only measured using fetal brains extracted 24 h after maternal treatment due to loss of samples extracted 6 h later. However, the EtOH-mediated decrease in *Esr2* mRNA and ESR2 protein levels in *Ogg1* −/− (but not +/+) males (but not females) suggests that OGG1 may also sex-dependently regulate ESR2-mediated gene transcription, which has not been previously reported and merits further investigation.

### 4.5. OGG1- and EtOH-Dependent Effects on Behavioural Abnormalities

Previous studies that measured open field activity in untreated OGG1-deficient mice did not find differences at 3 months of age (sexes combined and tested at 20 lux) [50] or in 4-month-old male *Ogg1* −/− mice (lighting conditions not provided) [17]. However a decreased performance in an open field box test (measured via number of crossed lines, number of rears, mean speed and time immobile) was observed in aging (26 months) *Ogg1 −*/*−* mice (sexes combined and tested at 20 lux) [50]. Our own data using 3-month-old untreated OGG1-deficient mice showed no differences in open field activity except for a trend for increased time spent in the centre zone by *Ogg1* −/− females when tested at ~50 lux [10].

Herein, sex-dependent differences were observed in the saline-exposed group in our studies. This could be due to several factors that varied across studies [51], including age (6 weeks in our study), testing time, size of the apparatus, centre zone size and lighting conditions (~130 lux in our study), as well as the effect of a single saline injection during pregnancy. Altered time spent in the centre zone without differences in the total distance travelled have been reported in mouse models reflecting altered anxiety-related behaviour without any effect on ambulatory ability [28]. In our study, saline-exposed OGG1-deficient males spent more time in the centre without any effect on the total distance travelled, suggesting reduced fear and anxiety in OGG1-deficient males, which was not significantly altered in EtOH-exposed *Ogg1* −/− males. Unlike males, saline-exposed OGG1-deficient females travelled a greater distance compared to *Ogg1* +/+ females, without exhibiting any differences in time spent in the centre zone, suggesting increased ambulatory activity without any effect on fear and anxiety in saline-exposed OGG1-deficient females. In contrast, with in utero EtOH exposure, although the total distance travelled in *Ogg1 +*/*+* and −/− females was unaffected, the time spent in the centre zone was decreased in *Ogg1* +/+ but not −/− females, suggesting that the EtOH-mediated increased fear and anxiety was OGG1-dependent.

Prenatal exposure to EtOH has been shown to induce anxiety-like behaviour in rats and mice [52,53]. ESR knockout mice exhibit altered open field activity at 10–12 weeks of age in mice, confirming a role for ESRs in altered open field activity [54]. Our results suggest that anxiety-like behaviour may be both an OGG1- and sex-dependent effect of EtOH, and may involve mechanisms such as estrogen receptors and the interaction between OGG1- and ESR-mediated gene regulation.

Although no differences were observed in social interaction in saline-exposed *Ogg1* mice, EtOH decreased velocity and track length in *Ogg1* +/− mice, with a similar non-significant trend in *Ogg1* −/− mice, but no effect in *Ogg1* +/+ mice. In the USV test, both the saline- and EtOH-exposed *Ogg1* +/− progeny exhibited similarly increased interaction-induced USVs compared to *Ogg1* +/+ mice, which was significant for EtOH, suggesting that the increase was due to a heterozygous loss of OGG1 rather than EtOH. The mechanism may involve ESR-mediated gene regulation, as one study reported that *Esr1*/*2* knockout male mice exhibited an abolition of sexual behaviour and decreased USVs in the presence of females, and a role for ESRs in regulating aggressive behaviour in male mice has been reported [55]. In addition, mice lacking either ESR1 or ESR2 exhibit decreased social behaviour [56,57], whereas ovariectomized female rats exposed to estrogen subcutaneously exhibited decreased USVs when introduced to a familiar cage mate after a week of separation, which may be due to estrogen-induced increased social memory [58]. These studies suggest a role for estrogen receptors in interaction-induced USV, which our results suggest may be OGG1-dependent.

The absence of a significant effect of EtOH or *Ogg1* genotype on startle response in the prepulse inhibition test may be due to the relatively low dose of EtOH, given there was a non-significant trend of an EtOH-dependent decrease in prepulse inhibition in *Ogg1* −/− but not +/+ males and a decrease in *Ogg1* +/+ but not −/− females.

## 5. Conclusions

This study demonstrated OGG1-dependent effects of in utero exposure of the fetal brain to physiological ROS levels, or to a single low dose of EtOH on epigenetic marks and gene expression in fetal brains, and postnatal behaviour, potentially relevant to neurodevelopmental disorders including FASD. In particular, OGG1 alone or in association with 8-oxoG may play a role in estrogen receptor-mediated gene regulation, which may be further altered by the ROS-enhancing effect of in utero EtOH exposure, possibly contributing to sex-dependent differences in some behavioural disorders observed in OGG1 mice. The relatively modest effects of EtOH observed herein suggest that follow-up studies using a higher dose of EtOH will be necessary to fully understand the effect of EtOH on behavioural abnormalities in OGG1-deficient mice and the role of OGG1 in estrogen receptor-mediated gene regulation, among other pathways, at higher levels of oxidative stress, where OGG1 may work differently to regulate estrogen receptor-mediated gene expression. Given the OGG1-dependent alterations in some epigenetic marks and behavioural outcomes in saline-exposed progeny, it is likely that OGG1 epigenetically alters the regulation of additional genes contributing to developmental disorders, including disorders not involving xenobiotic exposure.

## Figures and Tables

**Table 1:** Summary of OGG1-dependent changes in saline- and EtOH-exposed −/− or +/− *Ogg1* DNA repair-deficient fetal brains compared to +/+ control brains.

Gene/Protein	Time Post-Exposure (h)	Saline-Exposed	EtOH-Exposed (Compared to Saline-Exposed Progeny of the Same *Ogg1* Genotype, Unless Otherwise Stated)
Epigenetic Marks (Figure 2)			
H3K9ac	6	-	↑ *Ogg1* +/+ ↓ *Ogg1* −/− (vs. EtOH-exposed +/+)
	24	↑ *Ogg1* −/−	-
H3K9me3	6	-	↑ *Ogg1* −/−
	24	-	↑ *Ogg1* −/−
H3K27me3	6	-	-
	24	↑ *Ogg1* −/−	-
5-mC	24	↑ *Ogg1* −/−	-
5-hmC	24	-	-
Gene expression (Figure 3)			
*Tet1*	6	-	-
	24	-	↑ *Ogg1* −/−
*Hdac2*	6	-	-
	24	-	↑ *Ogg1* +/+
*Nlgn3*	6	-	↓ *Ogg1* −/−
	24	-	-
*Reln*	6	-	-
	24	-	↑ *Ogg1* +/+
Estrogen mRNA levels (Figure 4)			
*Esr1*	6	-	↓ *Ogg1* +/+ (M)
	24	-	↑ *Ogg1* −/− (combined sexes) ↑ *Ogg1* −/− (combined sexes, vs. EtOH-exposed +/+) ^1^
*Esr2*	6	-	↓ *Ogg1*−/− (M)
	24	-	-
*Esr1/2* ratio	6	-	↓ *Ogg1* +/+ (M)
	24	-	↑ *Ogg1* −/− (combined sexes) ↑ *Ogg1* −/− (F) (vs. EtOH-exposed +/+) ^1^
Estrogen Protein levels ^2^ (Figure 5)			
ESR1	24	-	-
ESR2	24	-	↓ *Ogg1* −/− (M) ↓ *Ogg1* −/− (M) (vs. EtOH-exposed +/+) ^1^
ESR1/2 ratio	24	-	-
*Esr1* loci select epigenetic marks (Figure 6)			
H3K27me3	6	-	↑ *Ogg1* +/+ (regions 2, 3, 4, 5)
H3K4me3	6	-	-
Behaviour (Figure 7 and Figure 8)			
Open field activity (total distance)	6 weeks	↑ *Ogg1 +*/− (F) ↑ *Ogg1* −/− (F)	
Open field activity (centre zone time)	6 weeks	↑ *Ogg1 +*/− (M)	↑ *Ogg1* −/− (F) (vs. EtOH-exposed +/+ and +/−) ↓ *Ogg1 +*/− (F)
Social interaction (velocity)	8 weeks	-	↓ *Ogg1 +*/− (combined sexes)
Social interaction (track length)	8 weeks	-	↓ *Ogg1 +*/− (combined sexes)
Female-induced USV	4–5 months	-	↑ *Ogg1 +*/− (compared to EtOH-exposed +/+)

Abbreviations: - = no change; M = males; F = females; USV = ultrasonic vocalization. ^1^ Significance level with this superscript: 0.05 < *p* < 0.1. ^2^ All other changes, at least *p* < 0.05 (see figures for specific levels). Protein levels were not determined at 6 h post treatment.

## Data Availability

The data presented in this study are available in this paper and the Supplementary Material.

## Supplementary Materials

The following supporting information can be downloaded at: https://www.mdpi.com/article/10.3390/cells12182308/s1, Figure S1: Histone modifications with no OGG1- or EtOH-dependent differences. Gestational Day (GD) 17 fetal brains exposed in utero to a single dose of EtOH (2 g/kg, i.p.) or its saline vehicle were extracted 1, 6 and 24 h later from *Ogg1* +/+ and -/- littermates and were assessed for histone modifications. No differences observed in H3K9ac (activation mark) and H3K9me3 (inactivation mark) at 1h and in H3K4me3 at 6 and 24 h (1 h not tested). The differences observed were determined using two-way ANOVA and a post-hoc Tukey’s test; Figure S2: List of genes with no OGG1- or EtOH-dependent differences in expression. Gestational Day (GD) 17 fetal brains exposed in utero to a single dose of EtOH (2 g/kg, i.p.) or its saline vehicle were extracted 6 and 24 h later from *Ogg1* +/+ and -/- littermates and were assessed gene expression. No differences were observed in mRNA levels of the listed genes using the primers shown in Figure S8. The differences observed were determined using two-way ANOVA and a post-hoc Tukey’s test. Figure S3: Controls to validate the ChIP-qPCR technique. Positive controls are *β-actin* and *Gapdh*, and the negative control is *Hbby* (hemoglobin subunit beta y). Associations of histone H3 (total histone), histone H3 lysine 9 acetylation (H3K9ac, activation mark), histone H3 lysine 27 trimethylation (H3K27me3, repressive mark), and RNA polymerase II (Pol II, activation mark) with *Esr1* gene were measured via ChIP-qPCR. The positive controls appropriately show enrichment for H3K9ac and Pol II (activation mark) and low levels for H3K27me3 (inactivation mark), as they are constitutively active. Conversely, the negative control appropriately shows a low or absent signal for H3K9ac and Pol II with some enrichment for repressive mark H3K27me3, as *Hbby* is not transcribed in the brain. Association of H3K27me3 and Pol II with *β-actin, Gapdh*, and the negative control *Hbby* were not measured in *Ogg1* -/- fetal brain (listed as N/A or not applicable). IgG represents the negative control isotype IgG. Above data represent 1 sample. Figure S5: EtOH-mediated trend for increased association of H3K4me3 with the *Esr1* gene in female *Ogg1* +/+ but not -/- fetal brains. Quantitative PCR was performed using five different sets of primers directed against various regions of the *Esr1* gene (Figure S9), and each of the regions were amplified after chromatin was immunoprecipitated using antibodies against H3K4me3 (active promoter) and histone H3 (control). The association of H3K4me3:H3 ratio was normalized to 1 % input in various regions of *Esr1* of fetal brains exposed in utero to saline or EtOH. The significance of differences was determined using two-way ANOVA and a post hoc Tukey’s test. Figure S7: OGG1- and EtOH-dependent effects on the startle response measured via prepulse inhibition. No differences were observed aside from the expected decibel dose-response relationship. Significance was determined using two-way ANOVA and a post hoc Tukey’s test. Figure S8: Primer sequences used to analyze mRNA levels of genes via RT-qPCR. Figure S9: Primer sequences used to analyze the association of histone modifications on various sites of the *Esr1* gene and control genes via ChIP-qPCR.
