# Supplementary material for: Altered Epigenetic Marks and Gene Expression in Fetal Brain, and Postnatal Behavioural Disorders, Following Prenatal Exposure of Ogg1 Knockout Mice to Saline or Ethanol
Source: Cells. 2023 Sep 19;12(18):2308. doi: 10.3390/cells12182308 (PMC10527575; doi:10.3390/cells12182308)
Supplement: Supplementary file 1 [file cells-12-02308-s001.zip › cells-2510233-supplementary.pdf]

16th September 2023

Cells

SUPPLEMENTARY ONLINE DATA

Altered epigenetic marks and gene expression in fetal brain and postnatal behavioural disorders following prenatal exposure of *Ogg1* knockout mice to saline or ethanol

Shama Bhatia[1,2], David Bodenstein[3,☆], Ashley P. Cheng[1,2,☆] and Peter G. Wells[1,2,3]

1. Dept. of Pharmaceutical Sciences, Faculty of Pharmacy, University of Toronto, Toronto, Ontario, Canada
2. Centre for Pharmaceutical Oncology, Faculty of Pharmacy, University of Toronto, Toronto, Ontario, Canada
3. Dept. of Pharmacology & Toxicology, Faculty of Medicine, University of Toronto, Toronto, Ontario, Canada

☆ These authors contributed equally

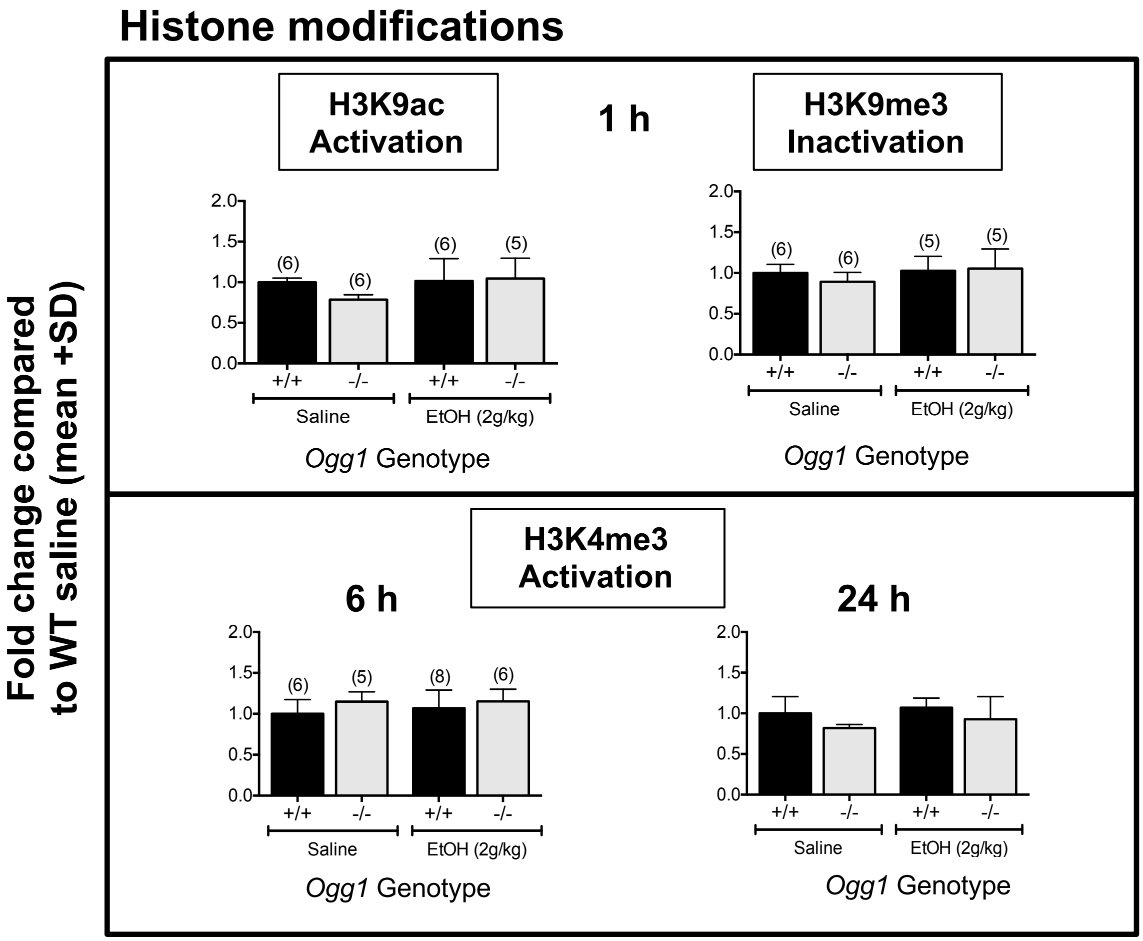

Figure S1. Histone modifications with no OGG1- or EtOH-dependent differences. Gestational Day (**GD**) 17 fetal brains exposed *in utero* to a single dose of EtOH (2 g/kg, i.p.) or its saline vehicle were extracted 1, 6 and 24 h later from *Ogg1* +/+ and -/- littermates and were assessed for histone modifications (1, 6 and 24 h). No differences observed in H3K9ac (activation mark) and H3K9me3 (inactivation mark) at 1h and in H3K4me3 at 6 and 24 h (1 h not tested). The differences observed were determined using two-way ANOVA and a post-hoc Tukey's test.

A list of genes with no OGG1- or EtOH-dependent differences in gene expression

Pathways	mRNA expression (no OGG1-dependent and EtOH-dependent differences)
**Epigenetic regulation**	Dnmt1, Dnmt3a, Hdac11, Mbd2, MeCP2, Smarca2, Tet2
**Neuronal communication, development and differentiation**	Drd2, Folr1, Kcne2, Nr2a, Nr2b, Maoa, Pdyn, Pnoc, Sema3b
**Tight junctions/cell adhesion/synapse**	Cldn2, Fmr1, Gabrb3, Ncam1, Shank3
**Antioxidative response/DNA Repair**	Nrf2, Parp1, Apex1
**OGG1-associated genes**	Hras, Nras, Otx2, RhoA, Sp1

Figure S2. List of genes with no OGG1- or EtOH-dependent differences in expression. Gestational Day (**GD**) 17 fetal brains exposed *in utero* to a single dose of EtOH (2 g/kg, i.p.) or its saline vehicle were extracted 6 and 24 h later from *Ogg1* +/+ and -/- littermates and were assessed gene expression. No differences were observed in mRNA levels of the listed genes using the primers shown in **Fig. S8**. The differences observed were determined using two-way ANOVA and a post-hoc Tukey's test. **Abbreviations: Apex1:** apurinic/apyrimidinic endodeoxyribonuclease 1; **Cldn2:** claudin 2; **Dnmt1:** DNA methyltransferase 1; **Dnmt3a:** DNA methyltransferase 3a; **Drd2:** dopamine receptor D2; **Fmr1:** fragile X mental retardation 1; **Folr1:** folate receptor 1; **Gabrb3:** gamma-aminobutyric acid type A receptor subunit beta 3; **Hdac11:** histone deacetylase 11; **Hras:** Harvey rat sarcoma viral proto-oncogene homolog encoding GTPase; **Kcne2:** potassium voltage-gated channel subfamily E regulatory subunit 2; **Maoa:** monoamine oxidase a; **Mbd2:** methyl-CpG binding domain 2; **MeCP2:** methyl-CpG-binding domain protein 2; **Ncam1:** neural cell adhesion molecule 1; **Nr2a:** *N*-methyl-D-aspartate receptor a; **Nr2b:** *N*-methyl-D-aspartate receptor b; **Nras:** neuroblastoma RAS viral oncogene homolog encoding GTPase; **Nrf2:** nuclear factor erythroid 2-related factor 2; **Otx2:** orthodenticle homeobox 2; **Parp1:** poly [ADP-ribose] polymerase 1; **Pdyn:** prodynorphin; **Pnoc:** prepronociceptin; **RhoA:** ras homology family member A; **Sema3b:** semaphorin-3B; **Shank3:** SH3 and multiple ankyrin repeat domains 3; **Smarca2:** SWI/SNF-related matrix-associated actin-dependent regulator of chromatin a2; **Sp1:** specificity protein 1; **Tet2**: ten-eleven translocation-2.

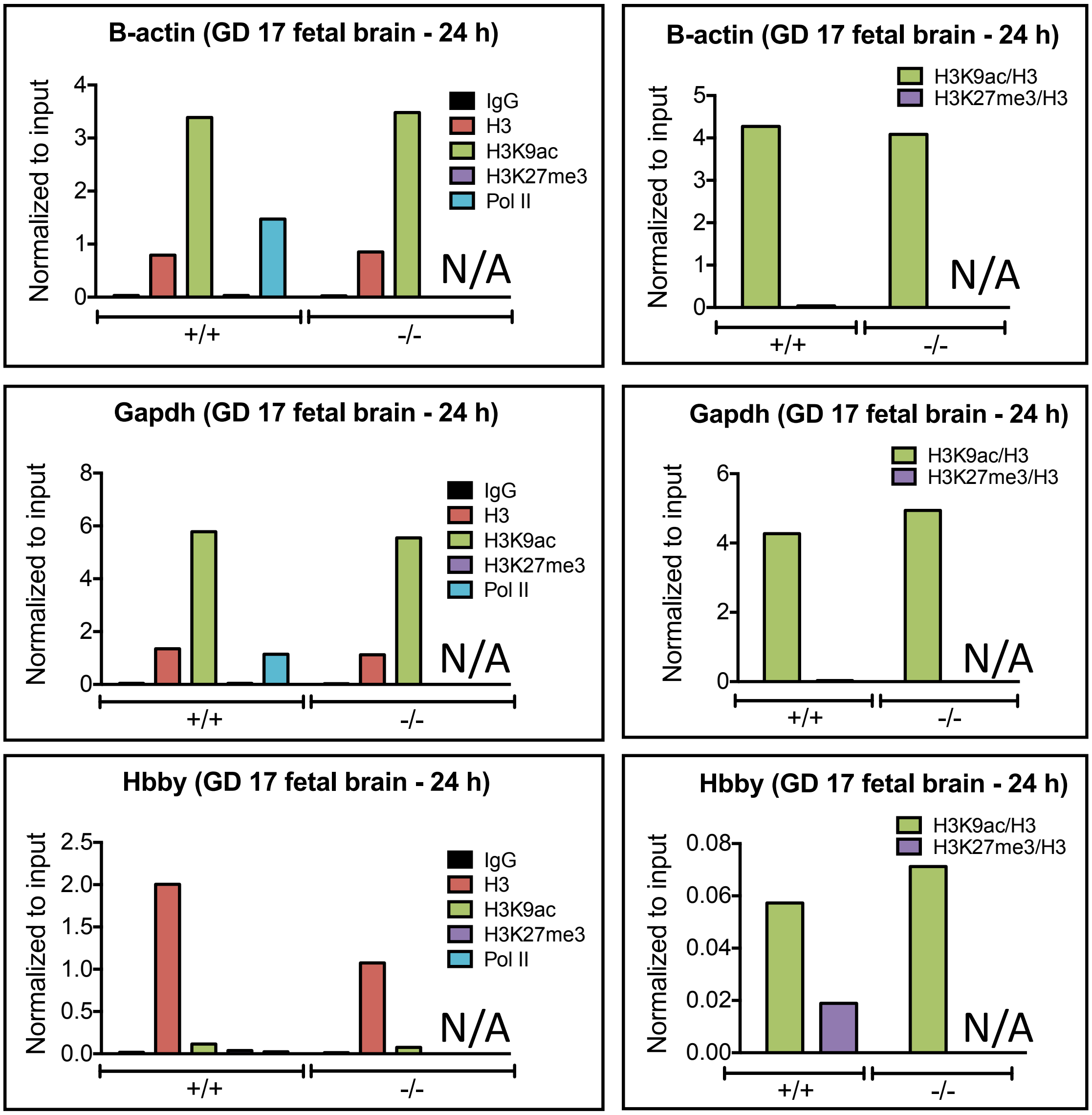

Figure S3. Controls to validate the ChIP-qPCR technique.

Positive controls are *β-actin* and *Gapdh*, and the negative control is *Hbby* (hemoglobin subunit beta y). Associations of histone H3 (total histone), histone H3 lysine 9 acetylation (**H3K9ac**, activation mark), histone H3 lysine 27 trimethylation (**H3K27me3**, repressive mark), and RNA polymerase II (**Pol II**, activation mark) with *Esr1* gene were measured via ChIP-qPCR. The positive controls appropriately show enrichment for H3K9ac and Pol II (activation mark) and low levels for H3K27me3 (inactivation mark), as they are constitutively active. Conversely, the negative control appropriately shows a low or absent signal for H3K9ac and Pol II with some enrichment for repressive mark H3K27me3, as *Hbby* is not transcribed in the brain. Association of H3K27me3 and Pol II with *β-actin*, *Gapdh*, and the negative control *Hbby* were not measured in *Ogg1* -/- fetal brain (listed as N/A or not applicable). IgG represents the negative control isotype IgG. Above data represent 1 sample.

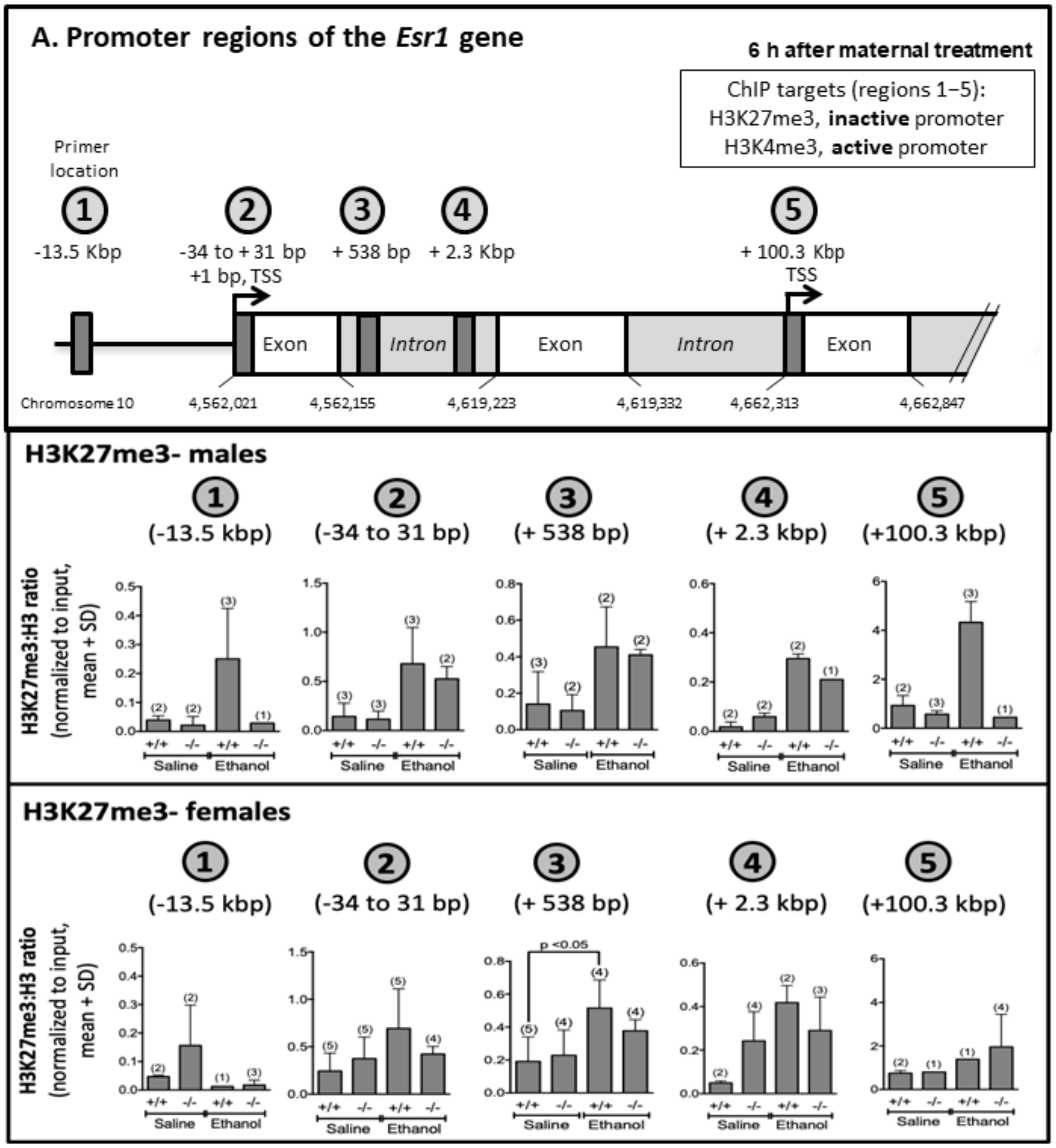

Figure S4. EtOH-mediated increased association of H3K27me3 with the *Esr1* gene in female *Ogg1* +/+ but not -/- fetal brains, with a similar trend in males. Quantitative PCR was performed using five different sets of primers directed against various regions of the *Esr1* gene (**Fig. S9**), and each of the regions were amplified after chromatin was immunoprecipitated using antibodies against H3K27me3 (inactive promoter) and histone H3 (control). The association of H3K27me3:H3 ratio was normalized to 1 % input in various regions of *Esr1* of fetal brains exposed *in utero* to saline or EtOH. The significance of differences was determined using two-way ANOVA and a post hoc Tukey's test.

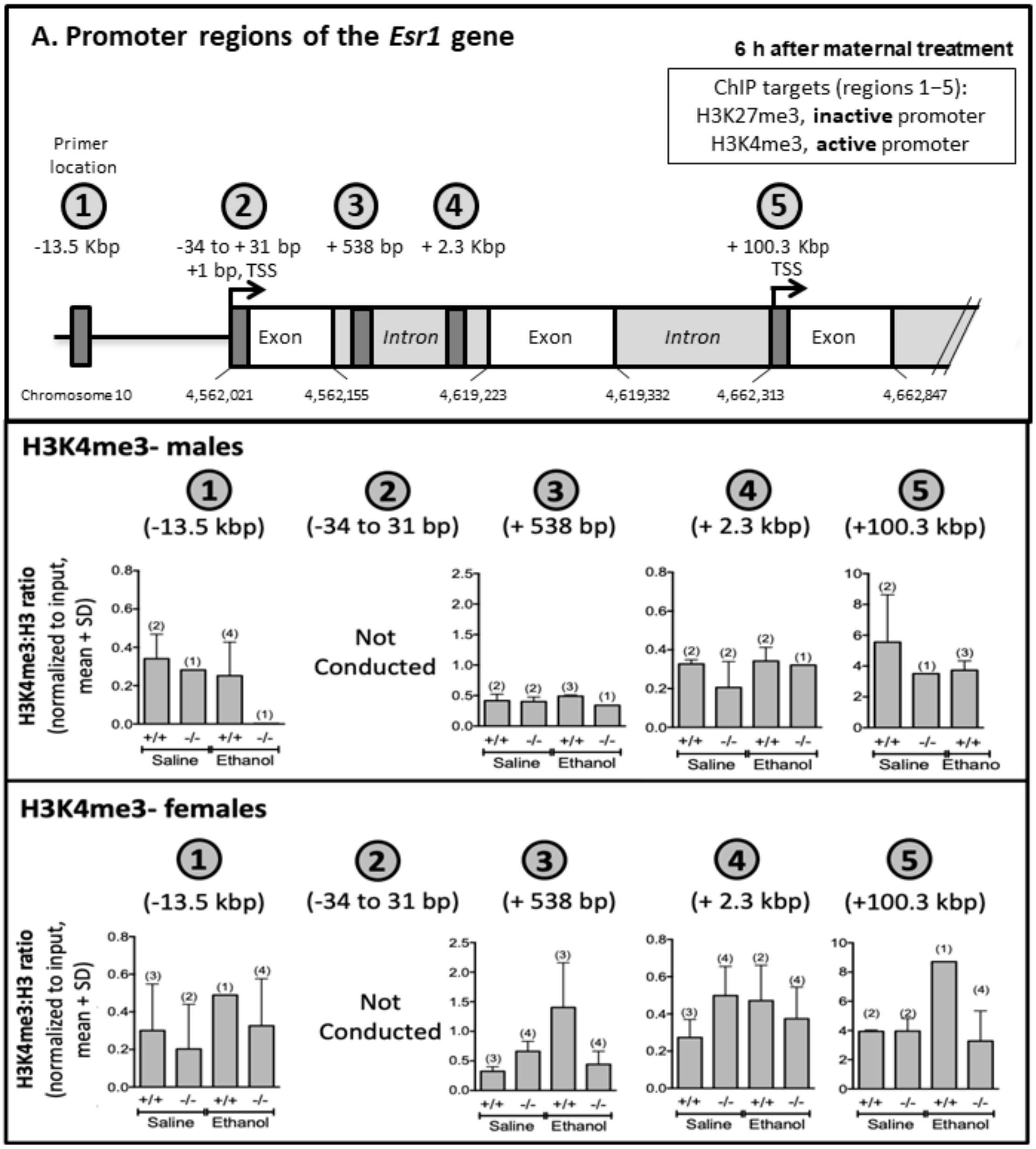

Figure S5. EtOH-mediated trend for increased association of H3K4me3 with the *Esr1* gene in female *Ogg1* +/+ but not -/- fetal brains. Quantitative PCR was performed using five different sets of primers directed against various regions of the *Esr1* gene (**Fig. S9**), and each of the regions were amplified after chromatin was immunoprecipitated using antibodies against H3K4me3 (active promoter) and histone H3 (control). The association of H3K4me3:H3 ratio was normalized to 1 % input in various regions of *Esr1* of fetal brains exposed *in utero* to saline or EtOH. The significance of differences was determined using two-way ANOVA and a post hoc Tukey's test.

A) Social interaction: measurements in the nonsocial zone

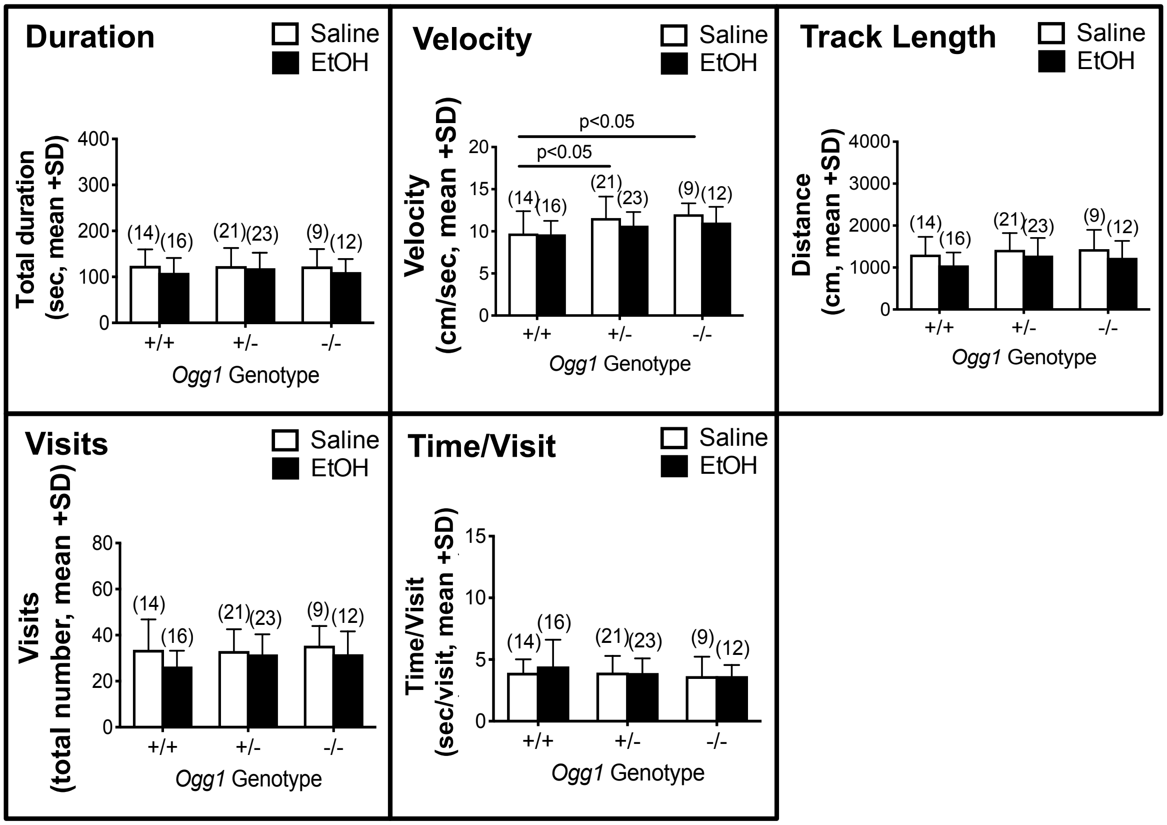

B) Female-induced ultrasonic vocalizations

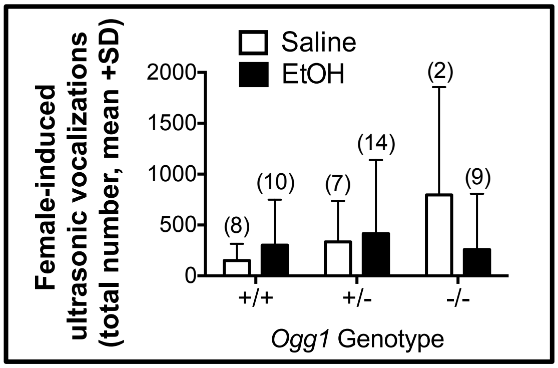

Figure S6: OGG1- and EtOH-dependent effects on social interaction and female-induced ultrasonic vocalizations. There were no sex differences, so male and female data were combined. **Panel A.** The test involved placing a mouse in a white walled arena of 62 x 40.5 x 23 cm with a novel social mouse placed inside one of the two inverted wire cups (defined as the social zone, see Fig. 7a), with the other cup placed empty (defined as the nonsocial zone, results shown above). In saline-exposed progeny, OGG1-dependent differences in social interaction were observed only for velocity. No differences were observed in EtOH-exposed progeny. **Panel B.** No differences observed in female-induced ultrasonic vocalizations in *Ogg1* mice, although the number of saline-exposed *Ogg1* -/- progeny was too low (n=2) to draw conclusions for that group. Significance was determined using two-way ANOVA and a post hoc Tukey's test.

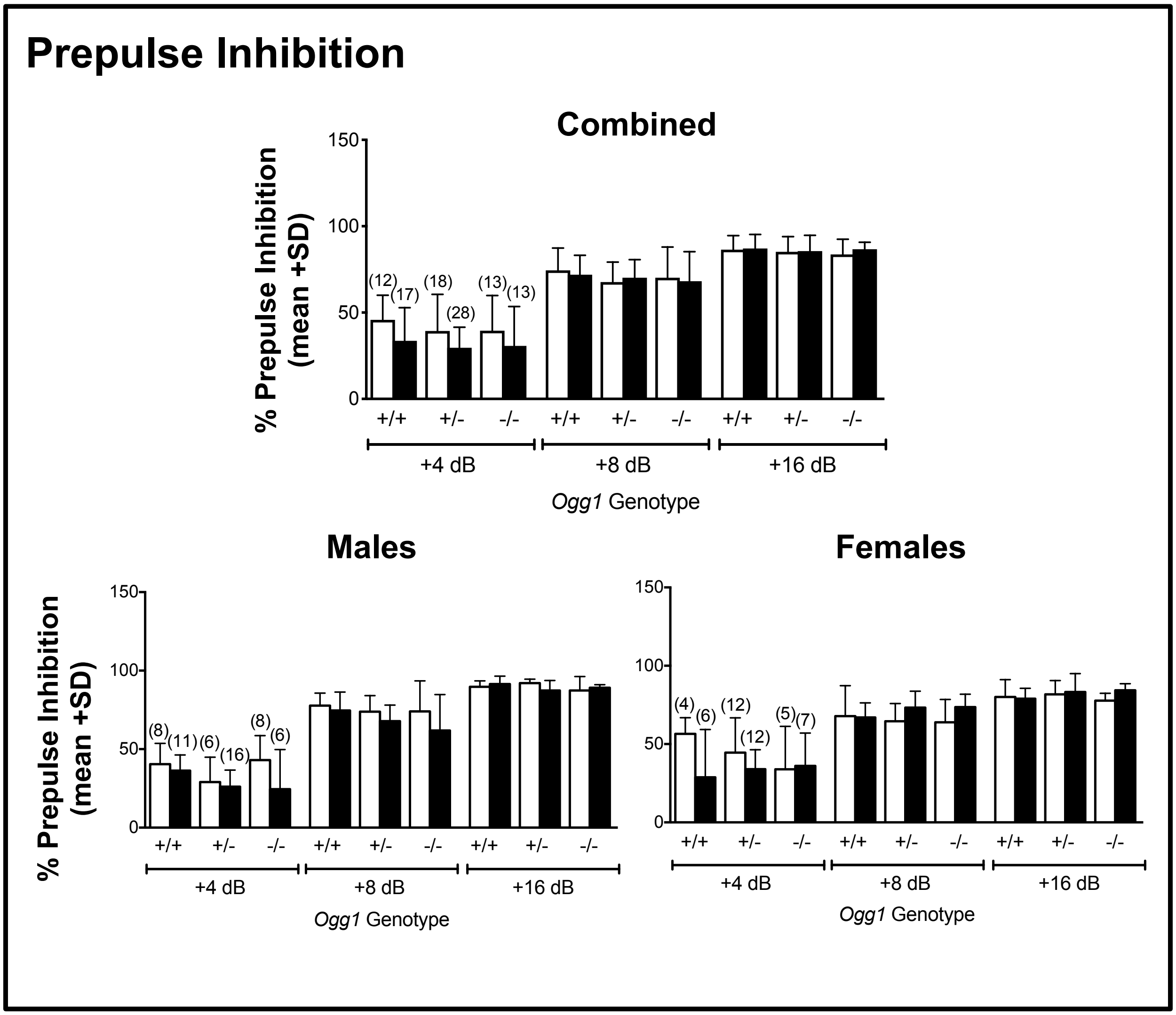

Figure S7: OGG1- and EtOH-dependent effects on the startle response measured via prepulse inhibition. No differences were observed aside from the expected decibel dose-response relationship. Significance was determined using two-way ANOVA and a post hoc Tukey's test.

Genes with OGG1- or EtOH-dependent differences

RT-qPCR Primers	Primer Sequence (5’ to 3’)
Esr1 Fwd	AAGTGTTACGAAGTGGGCATGA
Esr1 Rev	CCCTCCTCGGCGGTCTT
Esr2 Fwd	CTGTGCCTCTTCTCACAAGGA
Esr2 Rev	TGCTCCAAGGGTAGGATGGAC
Hdac2 Fwd	TTGATGGACTCTTTGAGTTTTGTCA
Hdac2 Rev	GTTTGTTGCCGGTTTAATTTCAC
Nlgn3 Fwd	GATTGCCTCTATCTGAATGTGTATGTG
Nlgn3 Rev	TCCTCGCCCTGTTTCTTAGC
Reln Fwd	TCACTGTGTCATACGCCAAGAAC
Reln Rev	GAGGTACAGGATGTGGATGACTGT
Tet1 Fwd	TTGAGGTCCTACAGCGGACAT
Tet1 Rev	TGGGTCGATGCCTTGACA

Genes with NO OGG1- or EtOH-dependent differences

RT-qPCR Primers	Primer Sequence (5’ to 3’)
Apex1 Fwd	TTTGTGCCGTGAGGGTCTCT
Apex 1 Rev	TGGCATCGCTGTGACGAA
Cldn2 Fwd	CCTTTCTCTGGACCTAGTCCTTGT
Cldn2 Rev	GCAGGCTCAAGAAGGCATCT
Dnmt1 Fwd	CACCTAGTTCCGTGGCTACGA
Dnmt1 Rev	CTCCTCTGCAGCCGACTCA
Dnmt3a Fwd	CGTCCGCAGCGTCACA
Dnmt3a Rev	GGGTTGACAATGGAGAGGTCAT
Drd2 Fwd	CCCTGGGTCGTCTATCTGGAG
Drd2 Rev	GCGTGTGTTATACAACATAGGCA
Fmr1 Fwd	GAGGATTGAGGCTGAAAATGAGA
Fmr1 Rev	TTATTGGAAGGTAGGGAACTTGGT
Folr1 Fwd	TGAGGACAATTTACACGACCAG
Folr1 Rev	TCCGATGTCATAGTTCCGCAG
Gabrb3 Fwd	GTCTGGTCTCCAGGAATGTTGTC
Gabrb3 Rev	CACCCACGAGAGGATTGTGA
Gapdh Fwd	CTCGTCCCGTAGACAAAATGG
Gapdh Rev	TGACCAGGCGCCCAAT
Hdac11 Fwd	TCACCACGTTACAACATCACCTT
Hdac 11 Rev	GCCATCGGACAGCAGCTT
Hras Fwd	TTTGCCATCAACAACACCAAGT
Hras Rev	CACCATTGGCACATCATCTGA
Kcne2 Fwd	GTGGATGCCGAGAACTTCTAC
Kcne2 Rev	CCACCACGATGAACGAGAAC
Mbd2 Fwd	AGACCCCCAGCGGATGA
Mbd2 Rev	CCTTGTAGCCTCTTCTCCCAGAA
MeCP2 Fwd	GCCGATCTGCTGGAAAGTATG
MeCP2 Rev	CATTAGGGTCCAAGGAGGTGTCT
Maoa Fwd	CAATTTCAATCACTCTGGATGACACTA
Maoa Rev	TTTAGCAAGTCGTTCAGCTTTCC
Ncam1 Fwd	TCATGTGCATCGCTGTTAACC
Ncam1 Rev	CCGTTCGGACCTCCACAAT
Nr2a Fwd	GTGGTGATCGTGCTGAATAAGG
Nr2a Rev	ATGCCGCAGGCTCAGAGT
Nr2b Fwd	CTATCCTGCAGCTGTTTGGAGAT
Nr2b Rev	GGCTGCTCATAACCTCATTCTTC
Nras Fwd	CCTCTACAGGGAGCAAATTAAGC
Nras Rev	CCAGTGTGTAAAAGGCATCCTC
Nrf2 Fwd	TCTCTGGAGGCAGCCATGA
Nrf2 Rev	CTGCTTGTTTTCGGTATTAAGACACT
Otx2 Fwd	ACCCGGTACCCAGACATCTTC
Otx2 Rev	GCGGCACTTAGCTCTTCGAT
Parp1 Fwd	CCCATCGACGTCAACTACGA
Parp1 Rev	CGTGGTAGCATGAGTGTTCTTCA
Pdyn Fwd	TGTGGCACTTCTCTGAGCTAGAGT
Pdyn Rev	CCCCCACGCAGATCTCAA
Pnoc Fwd	CCCTGCACCAGAATGGTAATG
Pnoc Rev	CCGGCCTGCAGGTCTTG
RhoA Fwd	ACGGGAGTTGGCCAAAATG
RhoA Rev	TGTTTGCCATATCTCTGCCTTCT
Sema3b Fwd	TCCCGCTGGCTCAATGAA
Sema3b Rev	GCGGACTCGCGGAAGAA
Shank3 Fwd	GACTGTGTGGAAGAAGTGCAGATG
Shank3 Rev	CGTCCGGTCCTCTCTGGTT
Smarca2 Fwd	GCCATCATTGATACTGTGATAAACTACA
Smarca2 Rev	TCCACTGGCTTCCTAATTAATTCAT
Sp1 Fwd	TCTGATCTCCAACCCCAAGCT
Sp1 Rev	CTTTGCCGCATCCTTGGAT
Tet2 Fwd	GTTTGGACTTCTCTGCTCATTCC
Tet2 Rev	CGACTTCTCGATTGTCTTCTCTATTG

Figure S8: Primer sequences used to analyze mRNA levels of genes via RT-qPCR.

ChIP-qPCR Primers	Primer Sequence (5' to 3')	Relative to Transcription Start Site (bp)	Amplicon product Size (bp)
**Controls**			
B-actin Fwd<br>B-actin Rev	CGGAGGCTATTCCTGTACATC<br>GCGAGAGAGAAAGCGAGATT	-260	91
Gapdh Fwd<br>Gapdh Rev	GCCACGCTAATCTCATTTTCTTC<br>TTCACACCGACCTTCACCATT	1126	69
Hbby Fwd<br>Hbby Rev	CTGACCCTCCCATGACCT<br>TCTGACCCTTTGTTCTGCAT	-134	90
***Esr1* Gene**			
Esr1 Fwd<br>Esr1 Rev	ACTGCTGAGCCACGGACTTAG<br>TGAGCGCTTCTCAGAGTTTCTCT	- 407	65
Esr1 Fwd<br>Esr1 Rev	TGGTGGTGCTAGCTCTTTGCT<br>CGAGGCTGTTATGACCATGAAAT	-34	65
Esr1 Fwd<br>Esr1 Rev	AACCGAGGTCTATGGAATGGAA<br>CCTCTCCACAGACACCGTGAA	-13.5 kpb	65
Esr1 Fwd<br>Esr1 Rev	TCCTGCTCCGACTTCACTTTG<br>ACAGCACCGAAGATTAAGAAACG	+538 bp	64
Esr1 Fwd<br>Esr1 Rev	CAGGTAGCTTTTGCTAAGGATGGT<br>AGAAAAAGGACTACTGGAACATTGC	+ 2.3 kbp	76
Esr1 Fwd<br>Esr1 Rev	GCGCTGCGCCTTCTCTAA<br>CGTGGGCCACCTGGAA	+ 100.3 kbp	53

Figure S9: Primer sequences used to analyze the association of histone modifications on various sites of the *Esr1* gene and control genes via ChIP-qPCR.
